# Convergence of 3D Bioprinting and Nanotechnology in Tissue Engineering Scaffolds

**DOI:** 10.3390/biomimetics8010094

**Published:** 2023-02-26

**Authors:** Shike Zhang, Xin Chen, Mengyao Shan, Zijuan Hao, Xiaoyang Zhang, Lingxian Meng, Zhen Zhai, Linlin Zhang, Xuying Liu, Xianghong Wang

**Affiliations:** 1 Henan Innovation Center for Functional Polymer Membrane Materials, Henan Key Laboratory of Advanced Nylon Materials and Application, School of Materials Science and Engineering, Zhengzhou University, Zhengzhou 450001, China; 2 National Engineering Research Center of Wheat and Corn Further Processing, College of Food Science and Engineering, Henan University of Technology, Zhengzhou 450001, China; 3 Henan Innovation Center for Functional Polymer Membrane Materials, Xinxiang 453000, China

**Keywords:** 3D bioprinting, nanobiomaterials, nanotechnology, tissue engineering scaffolds

## Abstract

Three-dimensional (3D) bioprinting has emerged as a promising scaffold fabrication strategy for tissue engineering with excellent control over scaffold geometry and microstructure. Nanobiomaterials as bioinks play a key role in manipulating the cellular microenvironment to alter its growth and development. This review first introduces the commonly used nanomaterials in tissue engineering scaffolds, including natural polymers, synthetic polymers, and polymer derivatives, and reveals the improvement of nanomaterials on scaffold performance. Second, the 3D bioprinting technologies of inkjet-based bioprinting, extrusion-based bioprinting, laser-assisted bioprinting, and stereolithography bioprinting are comprehensively itemized, and the advantages and underlying mechanisms are revealed. Then the convergence of 3D bioprinting and nanotechnology applications in tissue engineering scaffolds, such as bone, nerve, blood vessel, tendon, and internal organs, are discussed. Finally, the challenges and perspectives of convergence of 3D bioprinting and nanotechnology are proposed. This review will provide scientific guidance to develop 3D bioprinting tissue engineering scaffolds by nanotechnology.

## 1. Introduction

Tissue engineering (TE) is an interdisciplinary field that applies the principles of engineering and life sciences toward the development of biological substitutes that restore, maintain, or improve tissue functions [1]. The three elements of tissue engineering are scaffolds, cells, and signal molecules, of which scaffolds can be called “beating hearts” [2]. It is a 3D structure of an artificial extracellular matrix used in tissue engineering. It provides a temporary environment for cell adhesion, growth, reproduction, oxygen diffusion, nutrient transport, and waste removal, and minimizes inflammation and toxicity [3,4]. It can also be biodegraded at a controlled rate to support the formation of new tissues [5]. Based on the above, the scaffold needs to have (i) mechanical strength that can resist external pressure, maintain the original shape and integrity of the tissue, (ii) promote cell adhesion and proliferation, non-toxic, no inflammation reaction, and biocompatibility, (iii) appropriate degradation rate and other properties. Therefore, the chemical composition, physical structure, and biological functional parts are all important for tissue engineering scaffolds [6,7,8].

Considering the complexity of the human body, it is critical to select suitable biomaterials as scaffold media in tissue engineering. Currently, polymers (including natural polymers, synthetic polymers, and polymer-derived materials) have dominated tissue engineering scaffolds [9]. In parallel, nanotechnology is a broad, versatile, and diverse area and can be defined as the production, characterization, and application of different materials, devices, or systems while maintaining control of the shape and size on a nanometric scale [10]. Nanotechnology is excellent in constructing three-dimensional scaffolds suitable for various tissue engineering applications because its nanoscale size allows materials to support and guide cell activity at the cellular and subcellular levels. Therefore, nanofibers, nanoparticles, nanocrystals, and other types of nanomaterials are increasingly introduced as powerful tools for biofunctionalized scaffold materials to process and control the three-dimensional shape and geometry of scaffolds [11,12].

Choosing production technology depends on the specific requirements of the bracket, related materials, and machine constraints [13]. Various fabrication methods, such as freeze-drying, phase separation, gas foaming, particle leaching, and solvent casting, have been developed to produce tissue scaffolds in recent years. However, many manufacturing methods are traditional, not fully mimicking the inherent structure of the tissue or using organic solvents, and they are difficult to support cell growth. Among the technologies, 3D bioprinting is a promising strategy for fabricating scaffolds for tissue engineering. Based on an on-demand 3D model, it locates and assembles biomaterials (living cells can also be mixed in customized biomaterials) to manufacture biomedical products such as artificially implanted stents, tissues, and organs through computer-aided design software layered discretization and Computer Numerical Control molding methods. This technology not only has excellent control over the geometry and microstructure of the scaffold but also enables living cells and growth factors to be integrated into the scaffold during the manufacturing process [14].

This review first introduces the commonly used materials in tissue engineering scaffolds, both natural and synthetic, and reveals the improvement of nanomaterials on scaffold performance. Second, the 3D bioprinting technologies of inkjet-based bioprinting, extrusion-based bioprinting, laser-assisted bioprinting, and stereolithography bioprinting are itemized, and the advantages, the potential mechanisms are comprehensively stated from classification to application. Then the convergence of 3D bioprinting and nanotechnology applications in tissue engineering scaffolds, such as bone, nerve, blood vessel, tendon, and internal organs, are discussed. Finally, we discuss the challenges and perspectives in the development of convergence of 3D bioprinting and nanotechnology in tissue engineering scaffolds.

## 2. Materials for 3D Bioprinting

Nanotechnology is defined as the technological progress of basic nanoscale materials that can be applied in daily life [15,16]. As biomaterial-cell interactions are key to cell viability, proliferation, and differentiation, it is necessary to consider the characteristics of biomaterials, such as non-toxicity, good biocompatibility, and no immune and foreign body reaction [17]. In the process of using 3D printing technology to construct tissue engineering scaffolds, the use of nanomaterials enhances shape fidelity and printability apart from endowing plentiful biological functions to the bioinks [18]. In this part, 3D scaffolds are divided into synthetic polymer scaffolds, natural polymer scaffolds, and polymer derivative scaffolds according to the source of manufacturing materials. We discuss the integration of nanomaterials and 3D printing technology in these scaffolds. We summarize the advantages and disadvantages of the polymer in Table 1.

### 2.1. Natural Polymer

**Figure 1:**
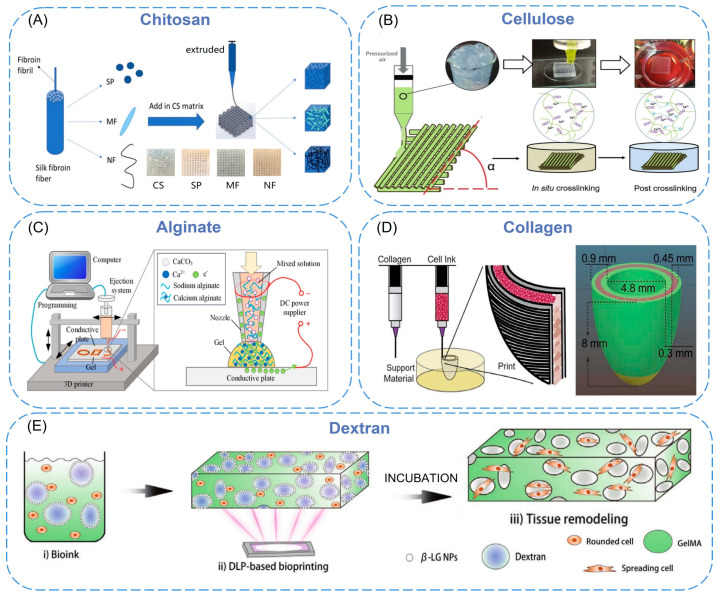
(**A**) Schematic elucidating 3D printing process of chitosan/silk composite scaffolds [28]. Copyright 2019, Elsevier. (**B**) Illustration of 3D printing of CNF hydrogels [38]. Copyright 2018, The Royal Society of Chemistry. (**C**) A schematic of the developed system: a PC is used to control the movement of the 3D printer and the ejection flow rate, and a DC voltage is applied across the conductive plate (anode) and the nozzle (cathode) to trigger the electrodeposition process [37]. Copyright 2017, IOP Publishing Ltd. (**D**) Schematic of dual-material printing using a collagen ink (green) and a high-concentration cell ink (pink) [34]. Copyright 2020, Elsevier. (**E**) Schematic illustration of DLP-based bioprinting of porous tissue constructs using nanoparticle-stabilized emulsion bioink [39]. Copyright 2022, Wiley.

Natural biopolymers have resurged over the past few decades as primary bioactive substances [19]. Biofunctional molecules which ensure bioactivity, biomimetic nature, and natural restructuring are typically found in such polymers. Natural polymers are macromolecular compounds existing in organisms, including chitosan [20], cellulose [21], alginate [22], and collagen [23] (Figure 1).

#### 2.1.1. Chitosan

The raw material source of chitosan is the exoskeleton of marine crustaceans, such as crabs, lobsters, shrimps, and krill, which has low toxicity, a changeable structure, abundant functional groups, and can be processed into various shapes and sizes by 3D printing [24]. Although it has many advantages in tissue engineering, low strength limits the application of chitosan [25]. Nano-sized materials were added to chitosan as fillers and dispersed in the whole matrix to resolve the problem of structural defects. Sadeghianmaryan et al. [26] developed a chitosan/sodium alginate/nano-hydroxyapatite scaffold by impregnating sodium alginate and nano-hydroxyapatite (nHA) into a printed chitosan scaffold with 3D printing technology. The compression test showed that the addition of nHA increased the elastic modulus of the scaffold, and the live/dead cell analysis showed that nHA enhanced cell viability and attachment. Chen et al. [27] developed a 3D-printable chitosan cryogel using difunctional polyurethane nanoparticles as the crosslinker. The cryogel was printed on a liquid cryodeposition manufacturing platform by a 3D printer and had similar properties to bulk cryogel, such as high compressibility, elastic recovery, and water absorption (≈3200%). The cell experiments showed that the 3D-printed chitosan cryogel scaffold was injectable and shape-restorable, which could provide good mechanical integrity for the proliferation of human adipose-derived adult stem cells and cartilage differentiation. Zhang et al. [28] developed chitosan/silk composite scaffolds using silk nanoparticles, silk microfibers, and silk nanofibers by an extrusion-based 3D printing method (Figure 1A). The surface of the scaffolds is hydrophilic, and the fabricated scaffolds can support stable cell growth. Among them, the use of silk nanoparticle geometry had a significant effect on the mechanical properties, and the 3D-printed scaffolds prepared with silk nanofiber fillers have the highest fibroblast proliferation rate and more elongated morphology. These findings indicate the potential of nanomaterials as ink fillers.

#### 2.1.2. Cellulose

Cellulose is a kind of β-1,4-linked glucopyranoside polymer, which is environment-friendly, renewable, biodegradable, biocompatible, non-toxic, and can be covalently linked to many bioactive molecules [29]. Cellulose nanofiber (CNF) can be obtained in two ways. The first method is to turn plant cellulose into CNF. The second is to synthesize BC nanofibers through bacterial fermentation of sugar [30]. Nanocellulose has a large surface area and high mechanical strength, which is mostly used for complex scaffold fabrication and to increase the stiffness of biomaterials [31]. Samfors et al. [32] developed a method to create channel structures within nanoporous bacterial cellulose (BNC) scaffolds that used a 3D printer to interconnect macroporosity and vessel-like lumens. This structure enabled endothelial cells to be arranged to form a vascular-mimicking network, and the microscopic nano-morphology of the scaffold increased oxygen and nutrient diffusion, which could be used to simulate the vascular system or other tubular tissues. Xu et al. [33] developed a 3D printing method to form double-crosslinked nanocellulose hydrogels and explored optimized printing and geometric design parameters to further adjust the stiffness of the printed scaffolds so that the mechanical strength of the scaffolds could be well-tuned in the 3 to 8 kPa range and promoted cell proliferation when stiffness increased within this range (Figure 1B). This correlation was first demonstrated by 3D-printed nanocellulose hydrogels.

#### 2.1.3. Alginate

Alginate is non-toxic, hydrophilic, biocompatible, and biodegradable. However, bioinks composed of alginate have shear thinning properties and reduced viscosity (lower viscosity provides less shear stress, thus reducing cell damage), which can cause printing difficulties when using extrusion 3D bioprinting techniques [34]. In order to improve alginate applicability for bioink, it is often functionalized by nanomaterials to obtain better printability. Abouzeid et al. [35] used sodium alginate and polyvinyl alcohol (PVA)-grafted cellulose nanofibers to fabricate hydrogel scaffolds with 3D printing. By varying the CNF content, they investigated how the viscosity of the hydrogel changes along with the shear rate at room temperature. The results showed the increase in CNF content leads to higher viscosity values, which is related to the tight entanglement between the nanofibers. When the shear rate is increased, all the as-prepared hydrogel scaffolds exhibit shear-thinning behavior with decreasing viscosity; this may be related to the typical breakdown of the hydrogel nanofiber network at high shear rates [36]. Shang et al. [37] proposed a mixed 3D printing and electrodeposition method of calcium alginate hydrogel. The specific method is as follows: the injection syringe is connected to the traditional 3D printer, sodium alginate, and CaCO_3_ nanoparticles are sprayed from the injector nozzle to the conductive substrate as fillers, then the Ca^2+^ released from CaCO_3_ particles via electric pressure that makes the alginate cross-linked to form calcium alginate hydrogel. By controlling the voltage, deposition time, movement, and other parameters of the 3D printer, various 3D structured alginate gels can be formed (Figure 1C).

#### 2.1.4. Collagen

Collagen mainly exists in the human extracellular matrix (ECM) and is the most abundant protein in mammals. It is responsible for maintaining the sustainability of the three-dimensional microstructure. It has stability and high biocompatibility [40]. Li et al. [41] prepared a bioink for 3D printing by dispersing nano-hydroxyapatite and deproteinized bovine bone into collagen. They fabricated a porous scaffold using a 3D printer to obtain the bone replacement material closest to the natural bone structure and composition. By evaluating the biocompatibility, it was found that the composite material was beneficial to the proliferation and differentiation of bone marrow mesenchymal stem cells. Chou et al. [42] combined fused deposition modeling and electrospinning techniques to embed poly(d,l-lactide-co-glycolide) (PLGA) in collagen nanofibrous membranes and integrated them onto polylactic acid (PLA) bone plugs. The in vivo effects of PLGA/collagen nanofibers and PLA bolts on tendon-bone healing was investigated on a rabbit bone tunnel model by histology and tendon pullout assays. They found PLGA/collagen nanofibrous membranes triggered more tendon-osseointegration in the lateral cortex, indicating that the composite polymer could effectively promote tendon remodeling in the rabbit model. In a recent report, an elliptical left ventricular model was printed by using a dual-material printing strategy [43]. Collagen bioink was used as the structural component of the inner and outer walls to provide sufficient structural integrity and maintain the expected geometric shape (Figure 1D). The central core area combined high-density cell bioink (cardiac muscle cells derived from human stem cells, etc.) to complete synchronous contraction and directional action potential transmission.

#### 2.1.5. Dextran

Dextran is a bacterial polysaccharide consisting essentially of α-1,6 linked glucopyranoside residues with a small percentage of α-1,3 linked residues. It has the advantages of biocompatibility, low toxicity, relatively low cost, and simple modification [44]. Wang et al. [45] proposed a dual-functional GelMAdextran aqueous two-phase emulsion bioink, simultaneously formulated with IL-4-loaded AgGNRs and MSCs. They found that the PGelDex bioinks exhibited excellent printability in DLP, conventional extrusion, and handheld extrusion bioprinting methods. Furthermore, this functional micropore-forming bioink could successfully suppress both Gram-positive and Gram-negative bacterial growth. The data also showed that the existence of IL-4 and MSCs promoted the differentiation of the M2 phenotype of macrophages, indicating that biological ink has a potential anti-inflammatory function. Tao et al. [39] produced bio-inks through the integration of GelMA solution and β-lactoglobulin (β-LG) nanoparticles/dextran solution mixture and printed tissue constructs using 3D bioprinting technology based on digital light processing (Figure 1E). After the ink is solidified, it is immersed in the culture medium to wash out the dextran so as to form pores in situ, allowing nutrients and oxygen to diffuse into the 3D-printed hydrogel construction. This mode allows precise and complex structure bioprinting. In addition, they proved that after subcutaneous implantation in nude mice, the 3D-printed porous structure trachea showed the ability to support the survival and maturation of chondrocytes and induced successful cartilage reconstruction in vivo.

### 2.2. Synthetic Polymer

**Figure 2:**
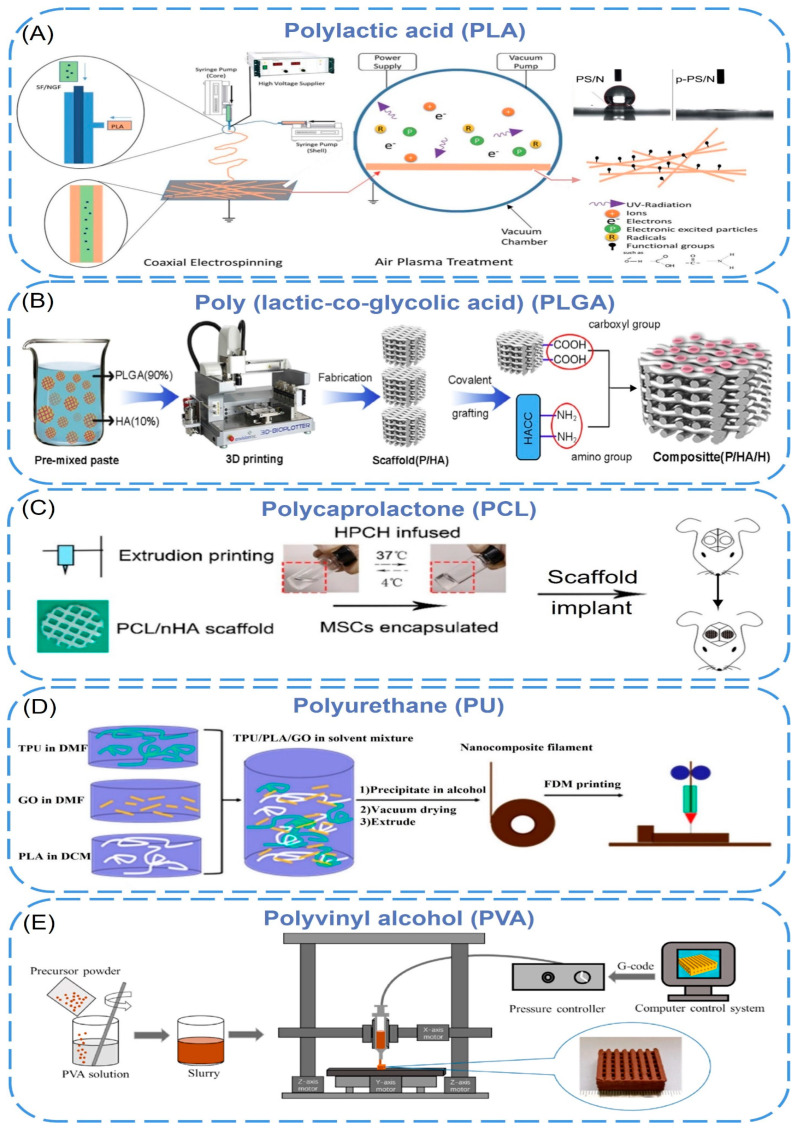
(**A**) Schematic illustration of the electrospinning and air plasma treatment towards the formation of p-PS/N scaffolds [55]. Copyright 2015, The Royal Society of Chemistry. (**B**) Schematic illustration for the synthesis mechanism of P/HA/H composite scaffolds [62]. Copyright 2016, Elsevier. (**C**) The schema of PCL hybrid scaffold fabrication and in vivo implant [67]. Copyright 2020, Ivyspring. (**D**) TPU/PLA/GO nanocomposites filament preparation and FDM printing process [64]. Copyright 2017, American Chemical Society. (**E**) Schematic diagram of 3DGP for preparing the porous scaffolds [69]. Copyright 2022, Elsevier.

Synthetic polymers are man-made polymers produced through chemical reactions with tunable chemical structures and physical properties. Compared to natural polymers, synthetic polymers are cheaper, strengthener, and have better functionality [46]. Some synthetic polymers are biodegradable, and these polymers can be degraded by microorganisms or biological fluids in vivo [47]. The commonly used biodegradable synthetic polymers include polylactic acid (PLA), poly(lactic-co-glycolic acid (PLGA), polycaprolactone (PCL), polyurethane (PU), and polyvinyl alcohol (PVA). However, few biodegradable synthetic polymers can meet all the needs of 3D printing of bioartificial organs. The current solution is combining these polymers with nanomaterials that are easy to be produced under controlled conditions and have strong mechanical properties [48] (Figure 2).

#### 2.2.1. Polylactic Acid (PLA)

Polylactic acid (PLA) is a biodegradable, bioabsorbable, thermoplastic aliphatic polyester. It is derived from renewable resources, contains repeating lactic acid units, and includes both D- and L-stereoisomers or be enantiomerically pure (e.g., PLLA contains only L stereocenters) [49,50,51,52]. However, the poor mechanical properties limited its applications [53,54]. To improve its mechanical strength, various nano-additives have been added to PLA. PLA-Silk Fibroin/NGF (PS/N) scaffolds were prepared by coaxial electrospinning, and p-PS/N nanofibers were obtained by air plasma treatment [55]. During coaxial electrospinning, PLA and silk fibroin nanofibers were sprayed by two different needles to form core-shell composite nanomaterials (Figure 2A). The diameters of PS/N and p-PS/N were 221 nm and 228 nm, respectively, and the water contact angle changed from 133.60° to 0°. It showed that plasma treatment significantly improved the hydrophilicity of the scaffold without damaging the fibers. In addition, the scaffold has been shown to support the attachment and differentiation of PC12 cells and can be used as a suitable matrix for neural tissue engineering. Naghieh et al. [56] fabricated several sets of pure PLA scaffolds with different pore sizes and shapes using fused deposition modeling (FDM), then developed the scaffolds by adding nanocomposite hairless gelatin forsterite layers via electrospinning technology. The formation of apatite on the scaffold surface was investigated by immersion in simulated body fluids. The results showed that the elastic modulus of the PLA/gelatin forsterite scaffold was significantly higher than the pure scaffold (about 52%), and the calcium phosphate-like precipitate formed on the surface confirmed that the nanocomposite fibrous layer had improved scaffold bioactivity. It is expected to be used for bone tissue regeneration in the future. In the study of K. Dave [57], amphiphilic nanomaterial carbon dots (CDs) were melt-blended with polylactic acid to be extruded into filaments suitable for 3D printing, and then they used FDM technology to print porous scaffolds, which were mixed with untreated materials. In contrast, treatments were found to enhance cell adhesion, proliferation, and migration in biological environments, providing opportunities for built-in scanning and monitoring of scaffolds and cellular environments. Prasopthum et al. [58] used extrusion-based 3D printing technology to incorporate hydroxyapatite nanoparticles (nHA) into polylactic acid (PLLA) to form nanofiber chains and then successfully printed self-supporting structures with different characteristics from nanometers to centimeters at room temperature. The mechanical properties of the scaffold were improved, and the osteogenesis of bone marrow mesenchymal stem cells was promoted.

#### 2.2.2. Poly(Lactic-co-Glycolic Acid) (PLGA)

Poly(lactic-co-glycolic acid) (PLGA) is a functional polymer organic compound, which is randomly polymerized from two monomers, lactic acid, and glycolic acid, and has adjustable biodegradability. However, the acidic microenvironment caused by its degradation products causes poor biological activity (e.g., osteoconductivity and osteoinductivity), so nano-materials are added to PLGA to improve this performance [59]. Rasoulianboroujeni et al. [60] introduced the advantages of 3D-printed PLGA/TiO2 nanocomposite scaffolds in re-bone tissue engineering. Compared with pure PLGA, the addition of TiO_2_ nanoparticles to PLGA not only increased the glass transition temperature, thermal decomposition onset point, and compressive modulus of the composite but also enhanced the surface wettability, which supported cell survival and increased bone tissue regeneration. Xia et al. [61] prepared the 3D-printed PLGA/HA/MgO (PHM) composite porous scaffolds by incorporating nano-hydroxyapatite (HA) and magnesium oxide (MgO) into a PLGA matrix. The experiments showed that, after adding inorganic components (HA and MgO), the composite scaffold could maintain its original pore structure throughout the degradation process with no significant change in surface morphology and showing a stable and slow degradation rate. What is more, the prepared PHM scaffold had good hydrophilic and mechanical properties similar to natural bone and a strong affinity for cell adhesion and proliferation, which can reduce inflammatory reactions and promote the formation of new bone. The porous scaffolds show a good application prospect in the field of bone repair. In another study [62], quaternary ammonium chitosan (HACC) was grafted onto a 3D printing scaffold composed of PLGA and hydroxyapatite to design a bone engineering scaffold with antibacterial and bone conduction properties (Figure 2B). The experiment proved that the combination of PLGA and HA enhanced the mechanical properties and bone conductivity of the scaffold. HACC reduced bacterial adhesion and biofilm formation in vitro and in vivo, and the scaffold generally showed good neovascularization and tissue integration.

**Table 1:** Key classes of materials discussed in this review and their key beneficial and detrimental properties.

Types	Polymers	Advantages	Disadvantages	Reference
Natural	Chitosan	Non-toxicity; Easy availability	Poor mechanical properties	[26,27,62]
	Cellulose	Adhesive and bioactive; Abundant and biodegradable	Mechanical stability lost during processing	[32,33,63]
	Alginate	Ease of use for 3D printing; Rapid gelation with divalent cations	Poorly adhesive; May damage cells during printing	[26,35,37]
	Collagen	Adhesive and bioactive; Abundant and biodegradable; Tolerant of functionalization	Mechanically weak; Contamination can lead to immunogenicity	[41,42,43]
	Dextran	Cost-effective; Biocompatibility	Low reproducibility due to variations in composition	[39,45]
Synthetic	Polylactic acid (PLA)	Degradable by hydrolysis; Properties dependent on monomer feedstock	Hydrolysis products may cause inflammation; Physically cross-linked gels are weak	[55,56,57,58,64]
	Poly(lactic-co-glycolic acid) (PLGA)	Adjustable biodegradability	Poor biological activity	[60,61,62]
	Polycaprolactone (PCL)	Degradable by hydrolysis; Stable hydrogels over wide concentration range	Insufficient mechanical strength; Crystallinity may slow hydrolysis beyond relevant timeframe	[65,66,67]
	Polyurethane (PU)	Biodegradability; High mechanical strength; Softness	Lack of cell adhesion sites; Less biocompatible	[63,64,68]
	Polyvinyl alcohol (PVA)	High elasticity; High biocompatibility and hydrophilicity	Non-degradable; Non-adhesive	[69,70,71]

#### 2.2.3. Polycaprolactone (PCL)

Polycaprolactone is a class of biodegradable aliphatic polyesters with insufficient mechanical strength. The incorporation of nanoparticles into the PCL matrix can improve the inherent properties of PCL. Jakus et al. [65] have compounded 90 wt% hydroxyapatite and 10 wt% polycaprolactones to form a liquid ink, which was printed by rapid 3D extrusion at room temperature into a synthetic bone regeneration biomaterial (HB). The obtained HB showed high elastic mechanical properties (~32 to 67% strain to failure, ~4 to 11 MPa elastic modulus) and absorbent (50% material porosity. Animal experiments proved that HB could be integrated rapidly with surrounding tissues to support new bone growth without the addition of biological factors. Yeo et al. [66] have proposed a PCL hierarchical structure. First, the PCL pillar was printed using a melt pressure of 3d; then micro/nanofibers were deposited on the surface of the PCL scaffold using an improved electrospinning method. Finally, cell-loaded bioinks (C_2_C_12_ myoblasts, polyethylene oxide, and alginate) were printed to the surface of the pillar. The experiments showed that the vertical PCL scaffolds provided excellent mechanical properties. The effective release and uniform distribution of cells in the scaffold were achieved by cell printing, and myoblast extension was induced by aligned micro/nanofibers. This printing method is expected to be used for muscle tissue regeneration. Ji et al. [67] have printed composite scaffolds by mixing nano-hydroxyapatite(nHA) into polycaprolactone and then implanted the hydroxypropyl chitin hydrogel embedded with mesenchymal stem cells to evaluate the osteogenic activity and mechanical properties (Figure 2C). The Young’s modulus of the printed PCL/nHA structure under compression was 2.34 ± 0.10 MPa and 2.33 ± 0.27 MPa, which was much higher than HPCH hydrogel (1.0 kPa). The co-culture experiment showed that the hybrid scaffold promoted macrophages to secrete growth factors, thereby promoting angiogenesis and osteogenesis.

#### 2.2.4. Polyurethane (PU)

The polyurethanes referred to here are thermoplastic linear polymers with biodegradability, high mechanical strength, softness, and high elasticity that can be processed into three-dimensional (3D) scaffolds by various fabrication techniques [72,73]. Chen et al. [64] blended thermoplastic polyurethane (TPU) with polylactic acid and added graphene oxide (GO) for fused deposition molding to achieve 3D printing of elastic TPU/PLA/GO nanocomposites (Figure 2D). They found that the addition of GO significantly improved the mechanical properties of the polymer matrix with a compressive modulus of 167% and a tensile modulus of 75.5%. The cell culture results showed that the 3D-printed scaffolds had good cell viability, which was conducive to cell proliferation, and had good potential as a tissue engineering scaffold. Chen et al. [63] prepared a novel high-viscosity, direct-printable polyurethane composite ink containing nanocellulose (CNF) and fused deposition 3D printing at low temperatures to fabricate various shapes and complex degree brackets. TEM images declared that CNFs were connected to multiple PU nanoparticles to form a “string” structure, and the continuous proliferation of fibroblasts was also proved in the scaffold by in vivo experiments. Hung et al. [68] printed a scaffold at low temperatures using polyurethane (PU) elastic nanoparticles, hyaluronic acid, and bioactive components, which were compliant and bioactive. When the scaffold was implanted into a rabbit cartilage defect, the release of bioactive components promoted the self-aggregation of mesenchymal stem cells (MSCs), which induced the differentiation of MSCs into chondrocytes and produced a cartilage repair matrix.

#### 2.2.5. Polyvinyl Alcohol (PVA)

Polyvinyl alcohol (PVA) is a synthetic polymer with good biocompatibility, and its viscoelastic properties are comparable to those of articular cartilage. With the addition of nano-additives, PVA scaffolds are an ideal matrix for tissue repair and regeneration [74]. H. et al. [69] prepared porous scaffolds by chemical coprecipitation and 3D gel printing technology. The printing paste based on polyvinyl alcohol and MgFe_2_O_4_ nanoparticles has a compressive strength between 1.8–12.51 mpa, which is suitable for human cancellous bone. A preliminary biological experiment showed that MC3T3-E1 cells had good adhesion and proliferation on the MgFe_2_O_4_ scaffolds surface, and MgFe_2_O_4_ scaffolds can promote ALP activation and calcium deposition, improving osteogenic ability. All this shows that MgFe_2_O_4_ magnetic scaffolds are suitable for cancellous bone repair (Figure 2E). Topsakal et al. [70] developed PVA-based 3D-printed composites as versatile biomaterials for orthopedic applications, where gold nanoparticles (AuNP) and ampicillin (AMP) were used for reinforcement. It was shown that the 3D-printed composite scaffolds based on PVA/AuNP/AMP had good biocompatibility, osteoinductivity, and antibacterial properties. Song et al. [71] fabricated 3D-printed scaffolds composed of nano-biphasic calcium phosphate (BCP), polyvinyl alcohol, and platelet-rich fibrin (PRF) at low temperatures with specific shapes and operable internal structures. The scaffold showed good bioactivity and biocompatibility both in vitro and in vivo. What is more, it also proved that PRF has the potential to enhance the repair of segmental bone defects in vivo.

### 2.3. Polymer Derivatives

Natural polymers are acquired from natural materials and provide an assurance of natural restructure, biomimetic nature, and bioactivity [75]. Natural polymer derivatives refer to more complex products which derived from the substitution of atoms or atomic groups of natural polymers. Generally, the function of a nano-filler is to solve a specific problem.

#### 2.3.1. Chitosan Derivatives

**Figure 3:**
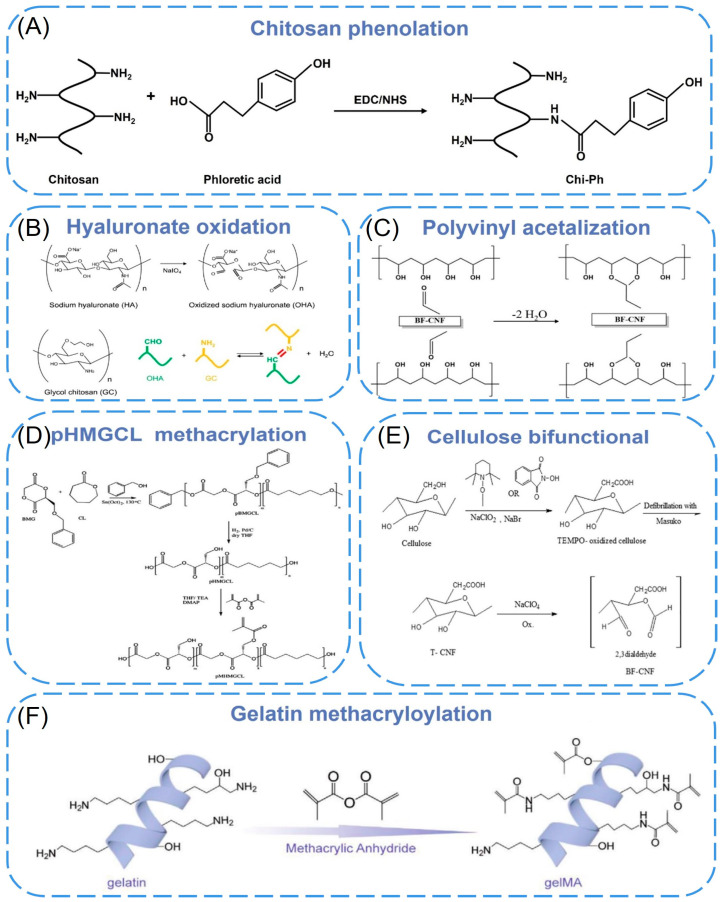
(**A**) Schematic illustration for conjugation of phloretic acid to chitosan using EDC/NHS chemistry [76]. Copyright 2021, Elsevier. (**B**) Chemical structure of OHA and GC, and Schiff base formation between OHA and GC [77]. Copyright 2019, Elsevier. (**C**) Chemical structure of carboxymethyl cellulose. (**D**) Functionalized pHMGCL/PCL with methacrylate and covalently grafted to gelMA hydrogel [83]. Copyright 2014, Elsevier. (**E**) Synthesis route for nanofibers with aldehyde and carboxylic acid groups [35]. Copyright 2020, Elsevier. (**F**) The reaction diagram of the synthesis of gelMA [81]. Copyright 2020, Wiley.

Chitosan has a changeable structure and abundant functional groups. The presence of a large number of amino groups allows chitosan to chemically react with anionic systems to change physicochemical properties. The use of functionalized chitosan to improve the self-healing properties of materials is the most commonly reported. Liu et al. [76] developed a chitosan self-healing hydrogel using modular 3D printing and secondary post-printing crosslinking, in which chitosan was functionalized with phenol (Chi-Ph) and then terminated with telespiral polyethylene glycol (DF-PEG) of benzaldehyde to form injectable hydrogel (CPDP) as printing ink (Figure 3A). The dynamic imine bond between amine on Chi Ph and benzaldehyde on DF-PEG, as well as the irreversible phenol bond between Chi-Ph chains, enable the hydrogel to have a faster gelling rate, higher modulus, better long-term stability, and self-healing ability. These properties may expand the biomedical applications of chitosan self-healing hydrogels. Ko et al. [77] selected ethylene glycol chitosan (GC) and oxidized hyaluronic acid (OHA) as basic materials, formed self-healing iron gel (GC/OHA/SPION) in the presence of superparamagnetic iron oxide nanoparticles (SPION), and then produced 3D structures through extrusion-based printing (Figure 3B). The reversible imine bond between OHA and GC is the key to inducing gel self-healing. Complete self-healing of GC/OHA/SPION ferrogel was observed at SPION > 5 wt%, and this autonomous healing was repeated several times, indicating that the gels were self-healing. Besides, by testing the viability of ATDC5 cells in the presence of polymer solutions or hydrogels, it was found that GC, HA, OHA, and GC/OHA/SPION gels did not show significant toxicity. It may have the potential as a magnetically actuated system in medicine, as it can stimulate and regulate cell differentiation in the presence of the magnetic field. Koosha et al. [78] prepared glyoxalylated chitosan/polyvinyl alcohol (PVA) hydrogel nanofibers by electrospinning in situ and introduced halloysite nanotubes (HNT) as a reinforcing agent, which increased the swelling degree from 272% to about 400%. Biocompatibility studies have shown that the presence of HNT increased the cells attached to the surface of the nanofibers, resulting in higher biocompatibility.

#### 2.3.2. Cellulose Derivatives

To improve the printability (rheological properties) and the formability of the nano-cellulose ink, nano-based auxiliary materials have been added to cellulose derivatives. The principle is to improve performance through particle-polymer interface interactions (electrostatic, van der Waals force, hydrogen bonds, covalent bonds, etc.) and energy dissipation. Xu et al. [38] demonstrated 3D printing and UV crosslinking of cellulose nanofibril (CNF)-based inks containing methacrylate derivatives. The CNF/gelatin methacrylate (GelMA) and CNF/galactomannan methacrylate systems were cross-linked with photoinitiators. By tuning the compositional ratio between CNF and GelMA, the compressive of Young’s modulus and local surface stiffness could be well tuned. The developed ink formulations are noncytotoxic and cytocompatible. Furthermore, the ink formula with CNF incorporated of GelMA, particularly with CNF/GelMA ratios of 2:1 and 5:1, promoted the proliferative activity of 3T3 fibroblasts in comparison with the plain CNF hydrogel. Kuzmenko et al. [79] proposed a conductive nanocellulose-based ink for 3D piezoelectric microvalve printing of neural scaffolds. The ink is composed of highly charged carboxymethyl nanocellulose (CNF) and water-based single-walled carbon nanomaterials (CNT) dispersions. The negatively charged deprotonated carboxyl groups on the fiber surface not only provided electrostatic repulsion between CNFs, but also CNTs could be better dispersed in water (Figure 3C). Cell culture studies showed that neural cells were preferred to attach, proliferate, and differentiate on CNF/CNT scaffolds. Cernencu et al. [80] designed a novel bio-based ink formulation suitable for 3D printing using pectin and carboxylated cellulose nanofibers. Rheological experiments demonstrated that the viscosity of the gel was determined by the nanofibers. The addition of pectin intensifies by increasing the viscosity drop, improving the printability of the ink formulation. The 3D-printed scaffolds based on pure CNF filaments presented a partial instability of the filaments that generate circular pores. The porous objects prepared with the new formulation showed a uniform shape and size of square-like pores, indicating that the bracket is uniform and stable after printing.

#### 2.3.3. Gelatin Derivatives

Gelatin, as a hydrophilic biomacromolecule, is denatured collagen with better processing properties than collagen, but gelatin does not have enough mechanical stability to support porous scaffolds. Gao et al. [81] used macromonomer gelatin methacryloyl chemically crosslinked gelatin (GelMA) to blend with nano-hydroxyapatite and combined with the sol-gel transformation (physical gel) of gelatin derivative aqueous solution to extrude a new physical hydrogel by 3D printing (Figure 3D). Itshowed that the porous bilayer scaffold composed of the hydrogel can induce osteochondral regeneration in vivo after implantation in mammalian joints. Pu et al. [82] printed GelMA/HAp scaffolds by direct ink writing using gelatin methacrylate anhydride (GelMA) with a high solid content of 82.5%, which was mixed with nano-hydroxyapatite (HAp) particles and anchored with functionalized polyphenol hydrogels. The scaffold and hydrogel were organically integrated into a biomimetic bone implant, which exhibited better water retention and mechanical stability than a single 3D-printed scaffold. It can promote the migration, proliferation, and osteogenic differentiation of bone marrow stem cells. Boere et al. [83] used poly(hydroxymethylglycolide-co-e-caprolactone)/poly(e-caprolactone)/PCL to functionalize thermoplastic polymer blends (pHMGCL/PCL) and grafted it onto gelatin methacrylamide (GelMA) hydrogel by photopolymerization (Figure 3E). Three-dimensional printed PHMGC/PCL and pMHMGCL/PCL scaffolds were used to enhance GelMA constructs and tested in a model of local articular cartilage defects. It was found that chondrocytes embedded in the constructs could form cartilage-specific matrices in vitro and in vivo. The results demonstrated that the embedded chondrocytes displayed significant cartilage-specific matrix deposition in these constructions.

## 3. Three-Dimensional Bioprinting Technology

Three-dimensional bioprinting is a promising method for constructing tissue engineering scaffolds. It is based on an on-demand 3D model. The file is discretely layered by computer-aided design software (CAD) and sent to a 3D printer [84]. After that, the biomaterial is partially assembled (living cells can also be mixed in customized biomaterials) to manufacture human scaffolds [5]. This technique not only provides excellent control over the geometry and microstructure of the scaffolds but also enables the incorporation of living cells and growth factors into the scaffolds during fabrication, which promotes the formation of biomimetic tissue [14].

According to the working principle, 3D bioprinting techniques can be divided into four categories [85]: (i) Inkjet-based 3D bioprinting; (ii) Extrusion-based 3D bioprinting; (iii) Laser-assisted bioprinting; (iv) Stereoscopic engraved bioprinting. Currently, these bioprinting modes have been widely used in the construction of tissue engineering scaffolds. We summarize the common characteristics of 3D printing technology in Table 2.

### 3.1. Inkjet-Based 3D Bioprinting

**Figure 4:**
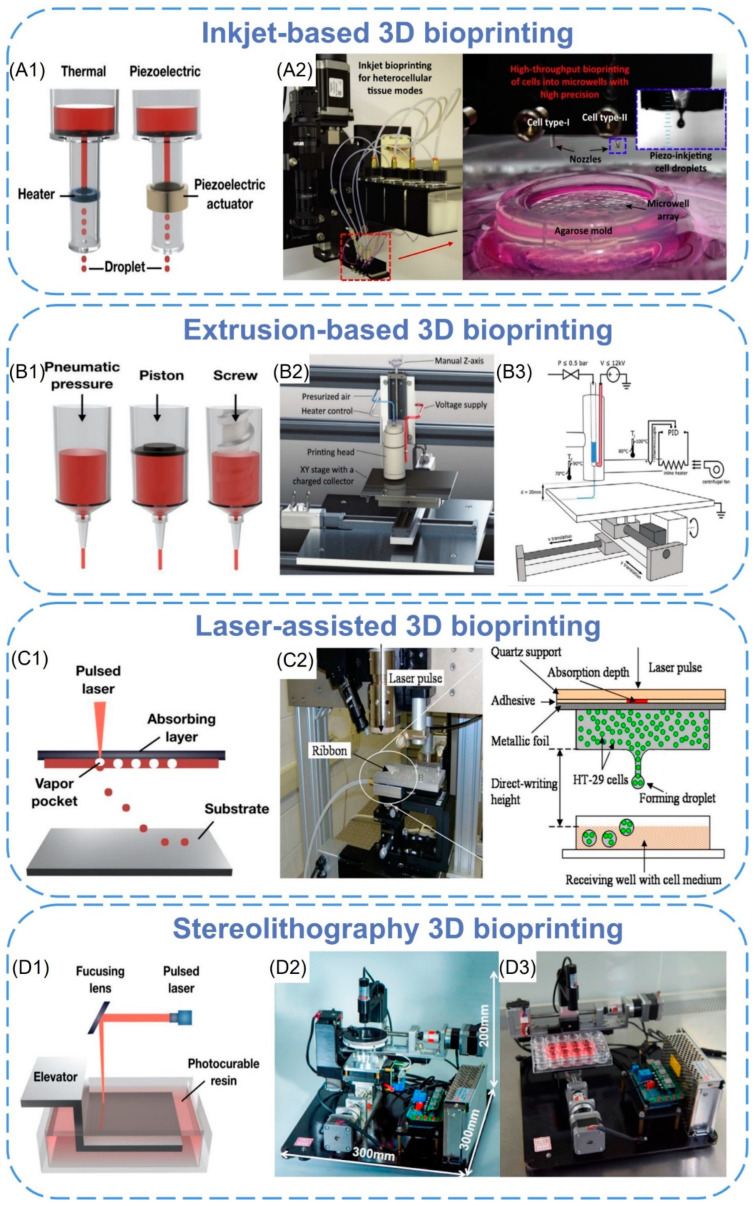
Bioprinting principles and devices. (**A1**) The mechanism diagram of the inkjet 3D bioprinting [85]. Copyright 2020, MDPI. (**A2**) A high-resolution inkjet-based bioprinter for fabrication of tissue models, where droplets of multiple cell suspensions are deposited into an agarose microwell array in a high-throughput manner [89]. Copyright 2016, Elsevier. (**B1**) The mechanism diagram of the extrusion-based 3D bioprinting [85]. Copyright 2020, MDPI. (**B2**,**B3**) Schematic of the MEW printer with the various components [96]. Copyright 2018, Wiley. (**C1**) The mechanism diagram of laser-assisted bioprinting [85]. Copyright 2020, MDPI. (**C2**) Experimental apparatus and schematic of proposed metallic foil-assisted laser cell transfer [105]. Copyright 2011, ASME. (**D1**) The mechanism diagram of the stereolithography bioprinting [85]. Copyright 2020, MDPI. (**D2**,**D3**) The stereolithography bioprinting device [106]. Copyright 2021, Elsevier.

Inkjet printing is the earliest bio-3D printing technology with a relatively low cost. The principle is similar to that of traditional 2D inkjet printing. Piezoelectric or thermally driven nozzles are used to divide the bioink (a mixture of hydrogel and cells) into a series of droplets (Figure 4(A1)). After layer-by-layer non-contact printing, a three-dimensional structure containing cells can be formed [86]. Cells and polymers can also be patterned into desired shapes by changing the content of the “ink” [87]. Park et al. [88] used inkjet printing to seed cells in patterns within collagen-containing bed to remodel the surrounding collagen matrices. Within the collagen matrices, fibroblasts rearranged and reorganized the surrounding ECM microenvironment, and ultimately, vertically elevated collagen microstructures were formed relevantly to the size and shape of the printed cell patterns. Finally, human skin models with papillary structures and the function of complex tissues at the dermo-epidermal junction were created.

According to the different printing technology, inkjet printing can be divided into continuous inkjet, drop-on-demand inkjet, and electrodynamic inkjet [89]. Continuous inkjet uses a piezoelectric driving device to fix pressure on the ink in the nozzle to make it continuously eject (Figure 4(A2)). The print speed of this continuous cycle inkjet system is high, and it can generate high-speed ink droplets, but it is rarely used in the field of bioprinting because of imprecisely [87].

In the drop-on-demand inkjet system, the ink is only ejected when needed for printing and deposited drop by drop in a “bottom-up” manner. For this shortcoming, the multi-nozzle method is often used to improve the printing speed [90]. Zimmermann et al. [91] developed a multicomponent inkjet bioprinting method by using a printing nozzle with two piezoelectric pipettes to dispense two reactive hydrogel solutions simultaneously, one of which contained human bone marrow-derived mesenchymal stem cells. The fabricated hydrogel scaffolds were shown to have cell-supporting properties. In addition, this technique may be used to control reducing tissue and organoid at a new overall level in the future.

An electric inkjet involves an electric field that uses micro-voltage changes to control the ejection of ink dots, which can precisely control the direction and shape of the ink dots. This technology is superior to other inkjet technologies because small-diameter orifices and high concentrations of bioink are used [92]. Electric inkjet 3D printing technology was applied with poly-lactic-co-glycolicacid by Tong Liu et al. to build biocompatible tissue engineering scaffolds [93]. By changing the electric field and drawing paths, different scaffold structures could be obtained, and these scaffolds were proven to be able to guide and improve cell proliferation and growth, potentially promoting wound healing.

Due to the small driving pressure of the nozzle, inkjet printing cannot print high-viscosity materials and high-density cells [94]. In addition, the low-viscosity material makes the structural strength smaller after printing, which cannot meet the needs of subsequent in vitro culture and transplantation. Therefore, the viscosity factor narrows the range of applicable biomaterials. What’s more, during the inkjet printing process, mechanical damage or thermal damage to cells may occur, and these disadvantages also limit the applications of inkjet printing technology.

### 3.2. Extrusion-Based 3D Bioprinting

Extrusion printing technology is the most widely used bioprinting method. This method uses pneumatic or mechanically driven nozzles to extrude biological ink in a controlled manner with a continuous process (Figure 4(B1)). Microfibers are extruded from the nozzles and deposited on the forming platform to form a two-dimensional structure. With the movement of the nozzle or the forming platform in the z-direction, the two-dimensional structure is stacked layer by layer to form a three-dimensional structure [95]. In the extrusion printing process, the continuous extrusion force can extrude uninterrupted fibers, not just single droplets. This forming method can print biomaterials with different viscosities. High-viscosity bioinks can provide better printability and structural integrity, and low-viscosity bioinks are more conducive to maintaining cell function. The material is suitable for a wide range of applications and can produce a structure with better structural strength.

The most common methods in extrusion-based 3D bioprinting are fused deposition modeling (FDM), melt electrowriting (MEW), and near-field electrospinning (NFES) [96]. In the FDM process, thermoplastic pellets or filaments of material are extruded by a temperature-controlled extruder and deposited layer by layer on a platform [97]. The direction of each deposited material layer and the spacing between material paths can be changed (i.e., layup mode), thereby obtaining scaffolds with a highly uniform internal honeycomb structure, controllable pore morphology, and complete pore interconnection [98]. Liu et al. [99] constructed a novel composite porous scaffold by combining large and small pores and honeycomb structures on FDM technology. It has a large pore size of 510 ± 20 μm and a small pore size of only 210 ± 15 μm. The scaffold owns significantly enhanced hydrophilicity, good mechanical properties, cell affinity, and osteogenic activity and would be an expected candidate as a bone repair material.

Melt electrowriting (MEW) combines melt electrospinning and 3D printing to provide higher-resolution printing. With the help of air pressure and an electric field, programmable electrospinning is realized by computer manipulation. Depending on the printing parameters, the resulting fiber diameter can be controlled, adjusted, and direct-written in different configurations [100]. Typically, fibers printed using MEW technology are in the range of several micrometers (2–50 μm) [96] therefore expanding the complexity and morphology of the resulting scaffolds (Figure 4(B2,B3)). Castilho et al. [101] designed and fabricated ultra-stretchable microfiber scaffolds with controllable hexagonal microstructures by melt electrowriting (MEW) technology, which exhibited up to 40% bidirectional deformations. The scaffold was combined with derived cardiomyocytes (induced pluripotent stem cells) in a collagen hydrogel to make a cardiac patch. It was found that its high-tension strain supports cardiac contraction and may be used for cardiac repair in the future.

Near-field electrospinning (NFES) is the extrusion printing method with the highest resolution, which has a resolution of less than 100 nm [102]. Based on the polymers soluble in volatile solvents, it creates 3D-aligned nanofiber scaffolds by using melts or solutions to control the deposition of electrospun fibers on mobile platforms [103]. Compared with MEW, it requires a higher voltage and a shorter distance between the nozzle and the collector plate. The printer setup is simpler because no heating element is required. However, this method has the risk of cytotoxicity due to the use of organic solvents, which limit cell encapsulation [98]. Fattahi et al. [104] reported an NFES method for the fabrication of controlled polymer fibers by printing polymethyl methacrylate fibers in cell-loaded 3D collagen gels for automation. The method controlled the deposition of single fibers precisely and fabricated high-resolution and repeatable 3D polymer fiber patterns. Fiber diameters were in the range of 1.86 ± 0.41 µm and aligned uniformly in the target direction. The results showed that the collagen gel on the printed fiber pattern exhibited good compatibility between cells and tissues. Besides, patterned fibers completely supported the hMSCs’ cellular growth.

### 3.3. Laser-Assisted 3D Bioprinting

Laser-assisted bioprinting (LAB) is also a headless inkjet printing method. The principle of LAB technology is that bioink is coated on the absorption layer on the transparent glass plate to form a three-layer structure of glass plate-absorption layer-bio-ink layer. Then pulse laser irradiation is focused on the absorption layer (Figure 4(C1)) so that a small part of the bioink layer at the irradiation position is vaporized and expanded. The bioink is squeezed from the surface to form a jet, which is then deposited on the receiving substrate [107]. Another similar technology is LIFT (last induced forward transfer); the difference is that the technique is written in continuous filaments [105,108] (Figure 4(C2)).

Compared with inkjet printing technology, this technology can avoid direct contact between bioink and processing devices, which does not cause mechanical shear damage to cells. This method can print higher-viscosity biomaterials, and the applicable range is wider than inkjet printing [109]. However, this technology is still mainly in the research stage and has few applications. The main reasons include (i) The printing cost is relatively high, and the process is not mature enough; (ii) It is time-consuming and labor-intensive when each layer is printed by bioink with the laser absorbing material coated; (iii) The repeatability and uniformity of the generated droplets need further research.

Most researchers used 1064 nm [110] and 193 nm [111] laser sources for bioprinting. Sorkio et al. [112] created a 3D corneal phantom by using human stem cells and laser-assisted bioprinting. Human embryonic stem cell-derived limbal epithelial stem cells were used as the cell source for printing epithelial-mimicking structures, while human adipose tissue-derived stem cells were used to construct lamellar matrix-mimicking structures. During the printing process, two bases with different laser wavelengths and appropriate absorber layers were built. The first layer was 1064 nm wavelength; this layer acted as a donor glass slide combined with a 60 nm thin gold absorber layer. The second layer was 2940 nm wavelength, which was suitable for the absorption maximum of water and the hydrogel absorbing layer. The 3D-printed matrix constructions were implanted into porcine corneal organ cultures, and it was found that the above two cell types both maintained good viability after printing.

### 3.4. Stereolithography 3D Bioprinting

Stereolithography (SLA) bioprinting is a solid free-form, nozzle-less 3D printing technology based on photosensitive polymer formula, which also manufactures 3D tissue structure layer by layer [34]. Compared with traditional bioprinting methods, SLA has the advantages of multiscale, high-resolution, and rapid printing of highly complex scaffolds (Figure 4(D1)). Furthermore, it is highly relevant to clinical applications because of the ability to fabricate implantable scaffolds with anatomically precise geometries, precisely controlled surface properties, and tunable physical or chemical properties.

Lighting sources play an important role in defining the performance of the SLA process. The resolution and precision of printed parts are often determined by the characteristics of the illumination source. In addition, the mechanical properties of the stent can also be tuned by controlling the light conditions [113]. Based on the illumination source, SLA 3D bioprinting systems can be divided into single-photon and multi-photon bioprinting. In single-photon methods, one high-energy photon is absorbed by a photoinitiator to generate free radicals. Visible light and UV-based SLA 3D bioprinting systems belong to this category. In multiphoton or two-photon methods, multiple low-energy photons are sequentially absorbed by photoinitiators in excited transition states to generate radicals, and infrared (IR) illumination is a common illumination source for two-photon bioprinting systems [114,115].

Lee et al. [116] fabricated a novel 3D biomimetic neural scaffold with a tunable porous structure and embedded aligned fibers. They used a technique that combined SLA and electrospinning to embed the microfibers into a 3D printing hydrogel scaffold under the irradiation of a solid-state ultraviolet (355 nm) laser, in which the microfibers were composed of polycaprolactone (PCL) and PCL. The scaffold significantly improved neural stem cell adhesion. Furthermore, highly aligned PCL/gelatin fibers within the scaffold improved neurite outgrowth and orientation control in primary cortical neurons. Zhang et al. [106] proposed a 3D bioprinting system based on lightweight stereolithography, which can be used for the 3D bioprinting of methacryloyl-modified hydrogels as photocrosslinking materials (Figure 4(D2,D3)). The system had an intelligent structural design, equipped with a visible laser emitter with a spot diameter of 1 mm, a wavelength of 405 nm, and a Petri dish. A temperature control platform was also installed below the printer slot to prevent hydrogel condensation. Only a relatively small amount of bioink (measured in milliliters) was consumed per print. By printing scaffolds loaded with mesenchymal stem cells (mBMSCs) and culturing mBMSCs on the scaffolds, it was found that the cells in the scaffolds were concentrated on the surface of the gel scaffolds, and the survival rate exceeded 95%. In addition, after five days, mBMSCs showed excellent survival on the GelMA scaffold (morphologically stretched, migrated, and exhibited a certain functional structure).

## 4. Three-Dimensional Bioprinting and Nanotechnology for Tissue Engineering Scaffolds

Three-dimensional bioprinting is a powerful technology with a remarkable ability to create three-dimensional scaffolds for tissue engineering and to control the geometry and microstructure of scaffolds. Nanotechnology is mainly used in tissue engineering and regenerative medicine. According to different purposes and functions, as well as its inherent characteristics and presentation in the biological environment, the technology creates many possibilities for generating highly complex cell structures. It paves the way for biological manufacturing with the integration of the two technologies by improving the key weaknesses of the manufacturing process and has great potential in the construction of tissue engineering scaffolds.

### 4.1. Bone Tissue Engineering Scaffold

Healthy bones are essential to maintain the important function of the human body. Bone defects, infection, tumors, and congenital diseases can cause patients to lose their basic motor ability and seriously affect their quality of life [117]. Although bone is a flexible organization with self-repair ability, its ability to fill the big defect is still complicated and limited [118]. Three-dimensional printing has the ability to control the bracket structure. Scaffold design is an important step in bone repair. This is because the features of the porous structure, including the porosity, pore size, and pore interconnectivity, have a great influence on their biological performance and mechanical properties [119]. The use of nanoparticles for 3D printing bone tissue engineering brackets can further enhance its functionality.

**Figure 5:**
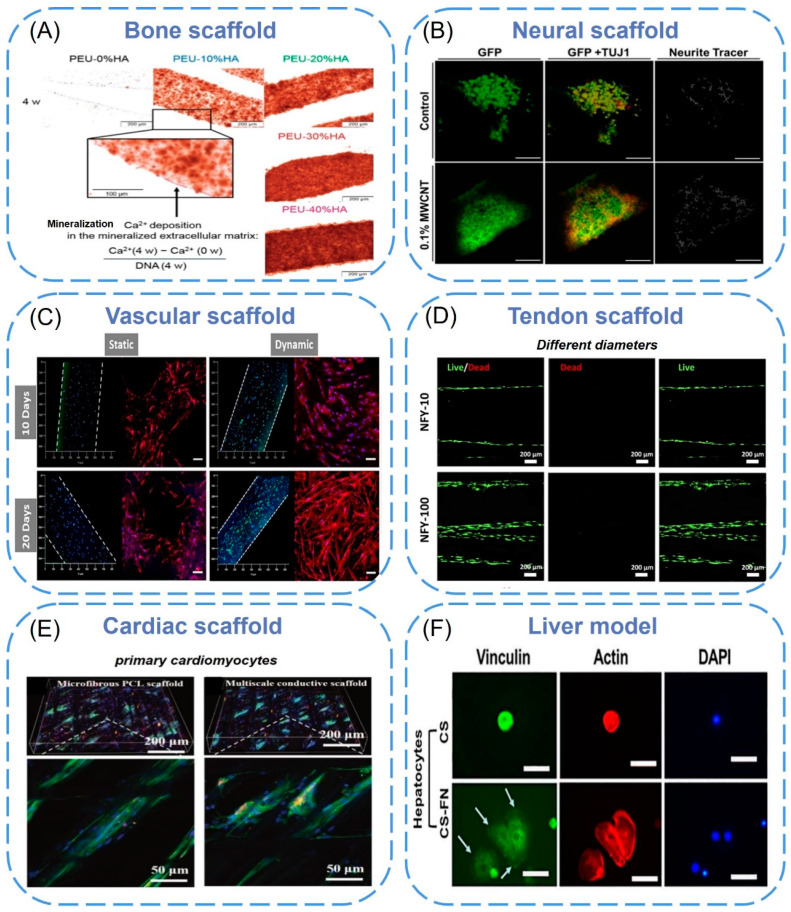
(**A**) Ca^2+^ deposited in the mineralized ECM on the surface of the filament in the scaffolds as visualized by Alizarin Red S staining. Dark red layers on the surface of the filaments in the bright field microscope image indicated enhanced mineralization resulting from higher HA contents in the scaffolds [120]. Copyright 2017, American Chemical Society. (**B**) Confocal micrographs of neural stem cells growth and spreading on 3D printed scaffolds at day 8. Cells were stained with GFP (green) and TUJ1 (red) [123]. Copyright 2018, IOP Publishing Ltd. (**C**) hMSCs (bone scaffolds, right columns) and HUVECs (blood vessel scaffolds, left columns) co-cultured, where the white dash lines indicate blood vessel channels [127]. Copyright 2021, Elsevier. (**D**) Fluorescence imaging of live (green) and dead (red) TSPCs that were seeded on aligned NFYs and cultured in a growth medium for 24 [128]. Copyright 2022, Frontiers Media S.A. (**E**) Immunofluorescence images of primary cardiomyocytes on the microfibrous PCL scaffolds and multiscale conductive scaffolds after eight days of culturing [133]. Copyright 2019, RSC Pub. (**F**) Vinculin focal adhesion and F-actin distribution of hepatocytes cultured on 2D chitosan or chitosan-FN film surfaces [134]. Copyright 2017, Wiley.

Yu et al. [120] blended 1,6-hexanediol L-phenylalanine poly(ester urea) (PEU) with hydroxyapatite (HA) nanocrystals through a fused deposition model and then printed it into a porous scaffold (porosity 75%). MC3T3-E1 preosteoblasts were inoculated in vitro to detect their biological activity. It proved that the mechanical properties of composite brackets (65~85 MPa) were significantly higher than polymer brackets made of other technologies (such as the salt soaking method) and other 3D printing pure polymer brackets. After four weeks of cell culture in the sample, the calcium concentration in the mineralization of the extracellular matrix increased by 185 times, indicating HA gave the bracket bone induction and bone transmission (Figure 5A). Nadi et al. [121] synthesized a strontium-doped boron iron ore nanoparticle and used 3D printing technology to prepare a new nanocomposite scaffold with polycaprolactone and polylactic acid to promote the regeneration of critical-sized bone defects. The scaffold was immersed in a simulated body fluid solution, and hydroxyapatite crystals were found on the surface of the scaffold. With the addition of nanoparticles, the formation rate of apatite increased, which proved that the scaffold supported the activity and proliferation of human osteoblasts. The nanocomposite scaffold was implanted into a critical-sized defect of the rat skull. After three months, it was found that the defect was filled with fibrous tissue, and osteocytes infiltrated into the implanted area to form blood vessels and a small amount of mineral deposition, implying that the scaffold could promote bone regeneration.

### 4.2. Neural Tissue Engineering Scaffold

As the nervous system is complex, it is still a challenge to repair damaged nerves and achieve full functional recovery. Because the extracellular matrix of human nerve tissue has a hierarchical structure from nanometer to micron, the nanostructured scaffold with bionic characteristics and excellent physical and chemical properties is very promising in nerve regeneration [122].

Lee et al. [123] combined multi-walled carbon nanotubes (MWCNT) with poly(ethyleneglycol) diacrylate (PEGDA) and used a stereolithography 3D printer with a 355 nm laser to fabricate MWCNT hydrogel nerve scaffolds easily, which have an adjustable microstructure and porous structure (Figure 5B). The results showed that the scaffolds combined with MWCNT promoted the proliferation of neural stem cells and neuronal differentiation. In addition, when the current is 500 μA, biphasic pulse stimulation can analyze protein expression by quantitative polymerase chain reaction and promote neuronal maturation. In another study [124], researchers used 3D printing technology based on stereolithography and coaxial electrospray to prepare a novel 3D biomimetic scaffold. To achieve nanostructure and sustained bioactive molecular delivery, the scaffold was embedded into core-shell nanoparticles with a continuous neurogenic factor delivery system. In addition, an adjustable porous structure was designed. The results showed that the scaffolds with higher porosity improved the adhesion of PC-12 cells significantly. More importantly, the improvement in the function of PC-12 and primary cortical neurons was observed on 3D printed nerve scaffolds containing bioactive factor nanoparticles, indicating its great potential in the repair and regeneration of peripheral nerve or central nervous system.

### 4.3. Vascular Tissue Engineering Scaffold

The vascular network is the basis for the continuous supplementation of oxygen and nutrition to cells. Vascular network 3D printing should have a specific level of angiogenesis ability to promote cell regulation, survival, growth, and differentiation. This emphasizes the biochemical characteristics of “biological ink” that combines 3D printing technology with various cellular hydrogels, which should be in line with specific engineering configurations [125].

Miao et al. [126] constructed a dynamic DNA hydrogel by using a nanoactivation strategy, namely vascular endothelial growth factor-modified black phosphorus nanosheets, and integrated it with a 3D-printed polylactic acid scaffold (VEGF-BP/DNA+PCL), giving the PCL scaffold a dual regulation of angiogenesis and skull regeneration bone. The black phosphorus nanosheets enhance the mechanical strength of dynamic self-healable hydrogel and endow the gel-scaffold construct with preserved protein binding to achieve sustainable delivery of growth factor. They found that the expression of angiogenic marker genes in the VEGF-BP/DNA+PCL construct was significantly up-regulated, and the ability of the construct to form HUVEC tubes in vitro was significantly improved, doubling the number of tubes, compared with the construct without VEGF. The results indicated that the VEGF-BP-DNA+PCL construct could significantly promote the angiogenesis performance of HUVEC. What is more, they confirmed that the gel-scaffold construct was able to promote the growth of mature blood vessels as well as induce osteogenesis to promote new bone formation. With the combination of SLA and FDM 3D printing systems, S.Y. Hann et al. [127] developed a biomimetic nanostructure with perfused endothelial vascular channels, in which nano-hydroxyapatite was incorporated into gelatinmethacryloyl bioink to form biological ink. The perfusion vascular channels were created in SLA-printed bone scaffolds by using FDM-printed polyvinyl alcohol templates. To predict the ability of vascular bone tissue to regenerate in vivo, human umbilical vein endothelial cells (HUVECs) were cultured in scaffolds (Figure 5C). The results showed that different capillaries sprouted through endothelial channels after the perfusion of HUVECs, indicating that the dual printing system was helpful in improving vascular growth.

### 4.4. Tendon Tissue Engineering Scaffold

Tendon tissue has a hierarchical structure and fewer blood vessels and nerves. The chaotic arrangement of new collagen in the scar tissue after injury will weaken the mechanical properties and function of the injured tendon. It is beneficial to combine 3D bioprinting and nanotechnology to develop a scaffold, which can promote the ability of tendon tissue to imitate the hierarchical structure of the natural tendon.

Yang et al. [128] arranged the three-dimensional oriented polycaprolactone nanofiber yarns (NFY) of different diameters neatly by dry-wet electrospinning. The NFY can simulate the hierarchical structure of collagen bundles and fibers in natural tendon tissue. By studying the effect of aligned NFYs on the differentiation of tendon stem/progenitor cells (TSPCs), it was found that the oriented nanofiber structure could induce tendon orientation and elongation, and the multi-scale structure of oriented NFY could improve tendon differentiation (Figure 5D). The results of rat tendon repair further showed that the bundled NFY promoted tendon repair in vivo by inducing new collagen tissue directionally. These data highlight its potential as a bionic multi-scale scaffold for tendon tissue regeneration. Wu et al. [129] developed a hybrid 3D porous scaffold, which consisted of an outer three-layer micron fiber bundle coiling from an electrohydrodynamic jet-printed polycaprolactone fiber network and an internal part making from a heat-sealed PCL tube under uniaxial tension. Compared with the rolled electrospun fibers, the cell metabolism cultured in tendon scaffolds was enhanced. In addition, the scaffold also led to the up-regulation of cell arrangement, cell elongation, and type I collagen formation.

### 4.5. Organs Tissue Engineering Scaffold

Although most studies have focused on bone, nerve, cardiovascular, and tendon tissue engineering, 3D bioprinting of other tissues has also been studied [130]. Biological imprinting of skin tissue applications (such as wound repair and dermis regeneration) requires structural enhancement, which is usually achieved by collagen nanoparticles or natural polymer mixtures [23,131]. Nanofiber cellulose is used to construct functional adipose tissue and to differentiate pluripotent stem cells into cartilage [132]. Recently, many scholars have tried to use 3D printing technology and nanotechnology to reconstruct tissue models of living tissues and finally make transplantable matrices for various organs.

Lei et al. [133] prepared multi-scale micro/nanofiber conductive scaffolds with layer-specific fiber orientation in a layer-by-layer manner by combining solution-based and melt-based electrohydrodynamic printing technology for cardiac regeneration. In each layer, eight PCL microfibers were reprinted and stacked vertically to form parallel microwells (Figure 5E). Compared with ordinary PCL scaffolds, multi-scale conductive scaffolds were found to guide the arrangement of cells between specific layers and significantly enhanced the expression of specific genes. Rajendran et al. [134] prepared chitosan nanofiber scaffolds by electrospinning and established a new three-dimensional liver model with a co-culture system composed of hepatocytes and fibroblasts (Figure 5F). Compared with the co-culture of single cell culture and three-dimensional culture system, it was found that the co-cultured hepatocytes formed colonies and maintained their morphology and function for a long time.

## 5. Conclusions and Future Directions

To sum up, this review focuses on 3D bioprinting technology and nanomaterials, as well as their latest development in tissue engineering scaffold integration, and highlights the main contributions of these nanomaterials. 3D bioprinting has made rapid progress in the manufacture of 3D functional biological structures. The application of nanotechnology and nanomaterials in the biomedical field has also made remarkable achievements. The synergistic combination of the two has proven to be an effective method for biological manufacturing processes, but there are still many challenges to be solved. For example, nanocomposite bioinks have been used to trigger specific cell reactions. However, the strict classification of target applications is not entirely clear. The highest resolution of printing reaches the micro range but is not enough to print at the capillary level, which is a long way to reproduce a complete and available biological organ. Scaffolds that bind cells and growth factors are difficult to store due to their low activity and instability. The distribution of three-dimensional cell microstructure and extracellular matrix in large three-dimensional scaffolds lacks spatiotemporal control, which limits their application in the regeneration of complex functional tissues (such as kidney and liver). Once the above problems are resolved, the development of 3D scaffolds may improve tissue regeneration and achieve wide application in the clinic.

## Figures and Tables

**Table 2:** Summary of common characteristics of 3D printing techniques.

	Inkjet-Based 3D Bioprinting	Extrusion-Based 3D Bioprinting	Laser-Assisted 3D Bioprinting	Stereolithography 3D Bioprinting
Applied Representative Materials	collagen, alginate, PEG	Alginate, GelMA, chitosan, PU	alginate, gelatin, PCL, PLGA	Photopolymers
Working Principle	Using thermal or acoustic force to eject very small size ‘bioink’ drops onto a substrate	Biomaterials are extruded though one or more nozzles under controlled pressure in a layer-by-layer pattern	Laser pulse generates a high-pressure bubble towards the collector substrate	A solid freeform, nozzle-free technology based on photosensitive polymer formulation under laser beam
Merits	High print resolution; Faster printing speed; Cell viability after printing; Low drive pressure;	Printable high-viscosity biomaterials; Wide range of printable biocompatible materials; More diversified material selection	No clogging of the cell/biomaterial; Does not cause mechanical damage to cells, thus increasing cell survival rates (above 95%); Multiple biomaterials can be used to print high-viscosity bioinks	Selective cross-linking of bioink using light and layer-by-layer solidification to for a 3D structure; High efficiency: Simpler device; Easy to control
Drawbacks	Cannot print high-viscosity materials or high concentration of cells; May cause mechanical or thermal damage to cells during printing; Lack of precision regarding droplet size and shape	Low print resolution; High mechanical and shear pressure stress; Relatively low cell viability	Complex control of laser printing systems; Few materials suitable for laser bioprinting; Side effects of laser irradiation on cells are not fully understood; Low printing efficiency	Ultraviolet light and its initiator can damage cells
Bioink Viscosity	3.5~12 MPa·s	30~6 × 10^7^ MPa·s	1~300 MPa·s	No limitation
Resolution	<70 μm	200–500 μm	10–100 μm	50 μm

## Data Availability

Not applicable.

## References

[1] Langer R., Vacanti J.P. (1993). Tissue Engineering. Science.

[2] Eivazzadeh-Keihan R., Maleki A., de la Guardia M., Bani M.S., Chenab K.K., Pashazadeh-Panahi P., Baradaran B., Mokhtarzadeh A., Hamblin M.R. (2019). Carbon based nanomaterials for tissue engineering of bone: Building new bone on small black scaffolds: A review. J. Adv. Res..

[3] Wang X., Shan M., Zhang S., Chen X., Liu W., Chen J., Liu X. (2022). Stimuli-Responsive Antibacterial Materials: Molecular Structures, Design Principles, and Biomedical Applications. Adv. Sci..

[4] Züger F., Berner N., Gullo M.R. (2023). Towards a Novel Cost-Effective and Versatile Bioink for 3D-Bioprinting in Tissue Engineering. Biomimetics.

[5] Nikolova M.P., Chavali M.S. (2019). Recent advances in biomaterials for 3D scaffolds: A review. Bioact. Mater..

[6] Liu X., Holzwarth J.M., Ma P.X. (2012). Functionalized synthetic biodegradable polymer scaffolds for tissue engineering. Macromol. Biosci..

[7] Wang X., Chen X., Song L., Zhou R., Luan S. (2020). An enzyme-responsive and photoactivatable carbon-monoxide releasing molecule for bacterial infection theranostics. J. Mater. Chem. B.

[8] Li X., Ding C., Li X., Yang H., Liu S., Wang X., Zhang L., Sun Q., Liu X., Chen J. (2020). Electronic biopolymers: From molecular engineering to functional devices. Chem. Eng. J..

[9] Gomes M.E., Rodrigues M.T., Domingues R.M.A., Reis R.L. (2017). Tissue Engineering and Regenerative Medicine: New Trends and Directions-A Year in Review. Tissue Eng. Part B Rev..

[10] Teleanu D.M., Chircov C., Grumezescu A.M., Teleanu R.I. (2019). Neurotoxicity of Nanomaterials: An Up-to-Date Overview. Nanomaterials.

[11] Gu B.K., Choi D.J., Park S.J., Kim M.S., Kang C.M., Kim C.H. (2016). 3-dimensional bioprinting for tissue engineering applications. Biomater. Res..

[12] Yang R., Wang X., Yan S., Dong A., Luan S., Yin J. (2021). Advances in design and biomedical application of hierarchical polymer brushes. Prog. Polym. Sci..

[13] Chia H.N., Wu B.M. (2015). Recent advances in 3D printing of biomaterials. J. Biol. Eng..

[14] Constante G., Apsite I., Alkhamis H., Dulle M., Schwarzer M., Caspari A., Synytska A., Salehi S., Ionov L. (2021). 4D Biofabrication Using a Combination of 3D Printing and Melt-Electrowriting of Shape-Morphing Polymers. ACS Appl. Mater. Interfaces.

[15] Neufurth M., Wang X., Wang S., Steffen R., Ackermann M. (2017). 3D printing of hybrid biomaterials for bone tissue engineering: Calcium-polyphosphate microparticles encapsulated by polycaprolactone. Acta Biomater..

[16] Li X., Liu B., Pei B., Chen J., Zhou D., Peng J., Zhang X., Jia W., Xu T. (2020). Inkjet Bioprinting of Biomaterials. Chem. Rev..

[17] Ouyang L., Yao R., Zhao Y., Sun W. (2016). Effect of bioink properties on printability and cell viability for 3D bioplotting of embryonic stem cells. Biofabrication.

[18] Peak C.W., Stein J., Gold K.A., Gaharwar A.K. (2018). Nanoengineered Colloidal Inks for 3D Bioprinting. Langmuir.

[19] Wang C., Yokota T., Someya T. (2021). Natural Biopolymer-Based Biocompatible Conductors for Stretchable Bioelectronics. Chem. Rev..

[20] Mallakpour S., Sirous F., Hussain C.M. (2021). Current achievements in 3D bioprinting technology of chitosan and its hybrids. New J. Chem..

[21] Lin K., Zhang D., Macedo M.H., Cui W., Sarmento B., Shen G. (2019). Advanced Collagen-Based Biomaterials for Regenerative Biomedicine. Adv. Funct. Mater..

[22] Rastogi P., Kandasubramanian B. (2019). Review of alginate-based hydrogel bioprinting for application in tissue engineering. Biofabrication.

[23] Lo S., Fauzi M.B. (2021). Current Update of Collagen Nanomaterials-Fabrication, Characterisation and Its Applications: A Review. Pharmaceutics.

[24] Ding C., Xu B., Zhang J., Sun Q., Chen Z., Liu S., Liu X., Chen J. (2021). Chitosan Wrapped Graphene/Polyurethane Composites with Improved Dielectric Properties for Capacitive Sensing. Polym. Sci. Ser. A.

[25] Bakshi P.S., Selvakumar D., Kadirvelu K., Kumar N.S. (2020). Chitosan as an environment friendly biomaterial—A review on recent modifications and applications. Int. J. Biol. Macromol..

[26] Sadeghianmaryan A., Naghieh S., Yazdanpanah Z., Alizadeh Sardroud H., Sharma N.K., Wilson L.D., Chen X. (2022). Fabrication of chitosan/alginate/hydroxyapatite hybrid scaffolds using 3D printing and impregnating techniques for potential cartilage regeneration. Int. J. Biol. Macromol..

[27] Chen T.C., Wong C.W., Hsu S.H. (2022). Three-dimensional printing of chitosan cryogel as injectable and shape recoverable scaffolds. Carbohydr. Polym..

[28] Zhang J., Allardyce B.J., Rajkhowa R., Kalita S., Dilley R.J., Wang X., Liu X. (2019). Silk particles, microfibres and nanofibres: A comparative study of their functions in 3D printing hydrogel scaffolds. Mater. Sci. Eng. C.

[29] Osorio D.A., Lee B.E.J., Kwiecien J.M., Wang X., Shahid I., Hurley A.L., Cranston E.D., Grandfield K. (2019). Cross-linked cellulose nanocrystal aerogels as viable bone tissue scaffolds. Acta Biomater..

[30] Lamm M.E., Li K., Qian J., Wang L., Lavoine N., Newman R., Gardner D.J., Li T., Hu L., Ragauskas A.J. (2021). Recent Advances in Functional Materials through Cellulose Nanofiber Templating. Adv. Mater..

[31] Lu Y., Yu J., Ma J., Wang Z., Fan Y., Zhou X. (2019). High-yield preparation of cellulose nanofiber by small quantity acid assisted milling in glycerol. Cellulose.

[32] Samfors S., Karlsson K., Sundberg J., Markstedt K., Gatenholm P. (2019). Biofabrication of bacterial nanocellulose scaffolds with complex vascular structure. Biofabrication.

[33] Xu C., Zhang Molino B., Wang X., Cheng F., Xu W., Molino P., Bacher M., Su D., Rosenau T., Willfor S. (2018). 3D printing of nanocellulose hydrogel scaffolds with tunable mechanical strength towards wound healing application. J. Mater. Chem. B.

[34] Li J., Wu C., Chu P.K., Gelinsky M. (2020). 3D printing of hydrogels: Rational design strategies and emerging biomedical applications. Mater. Sci. Eng. R Rep..

[35] Abouzeid R.E., Khiari R., Salama A., Diab M., Beneventi D., Dufresne A. (2020). In situ mineralization of nano-hydroxyapatite on bifunctional cellulose nanofiber/polyvinyl alcohol/sodium alginate hydrogel using 3D printing. Int. J. Biol. Macromol..

[36] Chimene D., Kaunas R., Gaharwar A.K. (2019). Hydrogel Bioink Reinforcement for Additive Manufacturing: A Focused Review of Emerging Strategies. Adv. Mater..

[37] Shang W., Liu Y., Wan W., Hu C., Liu Z., Wong C.T., Fukuda T., Shen Y. (2017). Hybrid 3D printing and electrodeposition approach for controllable 3D alginate hydrogel formation. Biofabrication.

[38] Xu W., Molino B.Z., Cheng F., Molino P.J., Yue Z., Su D., Wang X., Willfor S., Xu C., Wallace G.G. (2019). On Low-Concentration Inks Formulated by Nanocellulose Assisted with Gelatin Methacrylate (GelMA) for 3D Printing toward Wound Healing Application. ACS Appl. Mater. Interfaces.

[39] Tao J., Zhu S., Zhou N., Wang Y., Wan H., Zhang L., Tang Y., Pan Y., Yang Y., Zhang J. (2022). Nanoparticle-Stabilized Emulsion Bioink for Digital Light Processing Based 3D Bioprinting of Porous Tissue Constructs. Adv. Health Mater..

[40] Zheng J., Xie Y., Yoshitomi T., Kawazoe N., Yang Y., Chen G. (2022). Stepwise Proliferation and Chondrogenic Differentiation of Mesenchymal Stem Cells in Collagen Sponges under Different Microenvironments. Int. J. Mol. Sci..

[41] Li Q., Lei X., Wang X., Cai Z., Lyu P., Zhang G. (2019). Hydroxyapatite/Collagen Three-Dimensional Printed Scaffolds and Their Osteogenic Effects on Human Bone Marrow-Derived Mesenchymal Stem Cells. Tissue Eng. Part A.

[42] Chou Y.C., Yeh W.L., Chao C.L., Hsu Y.H., Yu Y.H., Chen J.K., Liu S.J. (2016). Enhancement of tendon-bone healing via the combination of biodegradable collagen-loaded nanofibrous membranes and a three-dimensional printed bone-anchoring bolt. Int. J. Nanomed..

[43] Lee A., Hudson A.R., Shiwarski D.J., Tashman J.W., Hinton T.J., Yerneni S., Bliley J.M., Campbell P.G., Feinberg A.W. (2019). 3D bioprinting of collagen to rebuild components of the human heart. Science.

[44] Naranda J., Bracic M., Vogrin M., Maver U. (2021). Recent Advancements in 3D Printing of Polysaccharide Hydrogels in Cartilage Tissue Engineering. Materials.

[45] Wang M., Li W., Luo Z., Tang G., Mu X., Kuang X., Guo J., Zhao Z., Flores R.S., Jiang Z. (2022). A multifunctional micropore-forming bioink with enhanced anti-bacterial and anti-inflammatory properties. Biofabrication.

[46] Ligon S.C., Liska R., Stampfl J., Gurr M., Mulhaupt R. (2017). Polymers for 3D Printing and Customized Additive Manufacturing. Chem. Rev..

[47] Kelly C.N., Miller A.T., Hollister S.J., Guldberg R.E., Gall K. (2018). Design and Structure-Function Characterization of 3D Printed Synthetic Porous Biomaterials for Tissue Engineering. Adv. Healthc. Mater..

[48] Rutz A.L., Hyland K.E., Jakus A.E., Burghardt W.R., Shah R.N. (2015). A multimaterial bioink method for 3D printing tunable, cell-compatible hydrogels. Adv. Mater..

[49] Wang G., Yang C., Shan M., Jia H., Zhang S., Chen X., Liu W., Liu X., Chen J., Wang X. (2022). Synergistic Poly(lactic acid) Antibacterial Surface Combining Superhydrophobicity for Antiadhesion and Chlorophyll for Photodynamic Therapy. Langmuir.

[50] Niu M., Wang H., Li J., Chen H., Li L., Yang H., Liu X., Chen Z., Liu H., Chen J. (2020). Polyethylene glycol grafted with carboxylated graphene oxide as a novel interface modifier for polylactic acid/graphene nanocomposites. R. Soc. Open Sci..

[51] Wang Y., Tian S., Sun Q., Liu W., Duan R., Yang H., Liu X., Chen J. (2019). Superhydrophobic Porous PLLA Sponges with Hierarchical Micro-/Nano-Structures for High-Efficiency Self-Cleaning. Macromol. Chem. Phys..

[52] Wang Y., Yang H., Liu H., Zhang L., Duan R., Liu X., Chen J. (2017). Controllable domain morphology in coated poly(lactic acid) films for high-efficiency and high-precision transportation of water droplet arrays. RSC Adv..

[53] Jing L., Yang H., Wang H., Zhang J., Liu S., Liu X., Liu W., Zhang L., Niu M., Chen J. (2020). Toughness Enhancement in Polyactide Nanocomposites with Swallow-Tailed Graphene Oxide. Polym. Sci. Ser. B.

[54] Liu H., Chen N., Shan P., Song P., Liu X., Chen J. (2019). Toward Fully Bio-based and Supertough PLA Blends via in Situ Formation of Cross-Linked Biopolyamide Continuity Network. Macromolecules.

[55] Tian L., Prabhakaran M.P., Hu J., Chen M., Besenbacher F., Ramakrishna S. (2015). Coaxial electrospun poly(lactic acid)/silk fibroin nanofibers incorporated with nerve growth factor support the differentiation of neuronal stem cells. RSC Adv..

[56] Naghieh S., Foroozmehr E., Badrossamay M., Kharaziha M. (2017). Combinational processing of 3D printing and electrospinning of hierarchical poly(lactic acid)/gelatin-forsterite scaffolds as a biocomposite: Mechanical and biological assessment. Mater. Des..

[57] Dave K., Mahmud Z., Gomes V.G. (2022). Superhydrophilic 3D-printed scaffolds using conjugated bioresorbable nanocomposites for enhanced bone regeneration. Chem. Eng. J..

[58] Prasopthum A., Shakesheff K.M., Yang J. (2018). Direct three-dimensional printing of polymeric scaffolds with nanofibrous topography. Biofabrication.

[59] Wang X., Sui S. (2011). Pulsatile culture of a poly(DL-lactic-co-glycolic acid) sandwiched cell/hydrogel construct fabricated using a step-by-step mold/extraction method. Artif. Organs.

[60] Rasoulianboroujeni M., Fahimipour F., Shah P., Khoshroo K., Tahriri M., Eslami H., Yadegari A., Dashtimoghadam E., Tayebi L. (2019). Development of 3D-printed PLGA/TiO2 nanocomposite scaffolds for bone tissue engineering applications. Mater. Sci. Eng. C.

[61] Xia X., Huang J., Wei J., Jin S., Zou Q., Zuo Y., Li J., Li Y. (2022). Magnesium oxide regulates the degradation behaviors and improves the osteogenesis of poly(lactide-co-glycolide) composite scaffolds. Compos. Sci. Technol..

[62] Yang Y., Yang S., Wang Y., Yu Z., Ao H., Zhang H., Qin L., Guillaume O., Eglin D., Richards R.G. (2016). Anti-infective efficacy, cytocompatibility and biocompatibility of a 3D-printed osteoconductive composite scaffold functionalized with quaternized chitosan. Acta Biomater..

[63] Chen R.D., Huang C.F., Hsu S.H. (2019). Composites of waterborne polyurethane and cellulose nanofibers for 3D printing and bioapplications. Carbohydr. Polym..

[64] Chen Q., Mangadlao J.D., Wallat J., De Leon A., Pokorski J.K., Advincula R.C. (2017). 3D Printing Biocompatible Polyurethane/Poly(lactic acid)/Graphene Oxide Nanocomposites: Anisotropic Properties. ACS Appl. Mater. Interfaces.

[65] Jakus A.E., Rutz A.L., Jordan S.W., Kannan A., Mitchell S.M., Yun C., Koube K.D., Yoo S.C., Whiteley H.E., Richter C.P. (2016). Hyperelastic “bone”: A highly versatile, growth factor–free, osteoregenerative, scalable, and surgically friendly biomaterial. Biomaterials.

[66] Yeo M., Lee H., Kim G.H. (2016). Combining a micro/nano-hierarchical scaffold with cell-printing of myoblasts induces cell alignment and differentiation favorable to skeletal muscle tissue regeneration. Biofabrication.

[67] Ji X., Yuan X., Ma L., Bi B., Zhu H., Lei Z., Liu W., Pu H., Jiang J., Jiang X. (2020). Mesenchymal stem cell-loaded thermosensitive hydroxypropyl chitin hydrogel combined with a three-dimensional-printed poly(epsilon-caprolactone) /nano-hydroxyapatite scaffold to repair bone defects via osteogenesis, angiogenesis and immunomodulation. Theranostics.

[68] Hung K.C., Tseng C.S., Dai L.G., Hsu S.H. (2016). Water-based polyurethane 3D printed scaffolds with controlled release function for customized cartilage tissue engineering. Biomaterials.

[69] Shao H., Wu J., Wang S., Duan J., Zhang Y., Peng J., Lin T. (2022). 3D gel-printing of porous MgFe2O4 magnetic scaffolds for bone tissue engineering. Ceram. Int..

[70] Topsakal A., Midha S., Yuca E., Tukay A., Sasmazel H.T., Kalaskar D.M., Gunduz O. (2021). Study on the cytocompatibility, mechanical and antimicrobial properties of 3D printed composite scaffolds based on PVA/ Gold nanoparticles (AuNP)/ Ampicillin (AMP) for bone tissue engineering. Mater. Today Commun..

[71] Song Y., Lin K., He S., Wang C., Zhang S., Li D., Wang J., Cao T., Bi L., Pei G. (2018). Nano-biphasic calcium phosphate/polyvinyl alcohol composites with enhanced bioactivity for bone repair via low-temperature three-dimensional printing and loading with platelet-rich fibrin. Int. J. Nanomed..

[72] Xu C., Hong Y. (2022). Rational design of biodegradable thermoplastic polyurethanes for tissue repair. Bioact. Mater..

[73] Wang Y., Wang L., Liu H., He S., Liu X., Liu W., Huang M., Zhu C. (2021). Polyurethane as smart biocoatings: Effects of hard segments on phase structures and properties. Prog. Org. Coat..

[74] Mansur H.S., Costa H.S. (2008). Nanostructured poly(vinyl alcohol)/bioactive glass and poly(vinyl alcohol)/chitosan/bioactive glass hybrid scaffolds for biomedical applications. Chem. Eng. J..

[75] Suamte L., Tirkey A., Babu P.J. (2023). Design of 3D smart scaffolds using natural, synthetic and hybrid derived polymers for skin regenerative applications. Smart Mater. Med..

[76] Liu Y., Wong C.-W., Chang S.-W., Hsu S.-H. (2021). An injectable, self-healing phenol-functionalized chitosan hydrogel with fast gelling property and visible light-crosslinking capability for 3D printing. Acta Biomater..

[77] Ko E.S., Kim C., Choi Y., Lee K.Y. (2020). 3D printing of self-healing ferrogel prepared from glycol chitosan, oxidized hyaluronate, and iron oxide nanoparticles. Carbohydr. Polym..

[78] Koosha M., Raoufi M., Moravvej H. (2019). One-pot reactive electrospinning of chitosan/PVA hydrogel nanofibers reinforced by halloysite nanotubes with enhanced fibroblast cell attachment for skin tissue regeneration. Colloids Surf. B Biointerfaces.

[79] Kuzmenko V., Karabulut E., Pernevik E., Enoksson P., Gatenholm P. (2018). Tailor-made conductive inks from cellulose nanofibrils for 3D printing of neural guidelines. Carbohydr. Polym..

[80] Cernencu A.I., Lungu A., Stancu I.C., Serafim A., Heggset E., Syverud K., Iovu H. (2019). Bioinspired 3D printable pectin-nanocellulose ink formulations. Carbohydr. Polym..

[81] Gao J., Ding X., Yu X., Chen X., Zhang X., Cui S., Shi J., Chen J., Yu L., Chen S. (2021). Cell-Free Bilayered Porous Scaffolds for Osteochondral Regeneration Fabricated by Continuous 3D-Printing Using Nascent Physical Hydrogel as Ink. Adv. Healthc. Mater..

[82] Pu X., Tong L., Wang X., Liu Q., Chen M., Li X., Lu G., Lan W., Li Q., Liang J. (2022). Bioinspired Hydrogel Anchoring 3DP GelMA/HAp Scaffolds Accelerates Bone Reconstruction. ACS Appl. Mater. Interfaces.

[83] Boere K.W., Visser J., Seyednejad H., Rahimian S., Gawlitta D., van Steenbergen M.J., Dhert W.J., Hennink W.E., Vermonden T., Malda J. (2014). Covalent attachment of a three-dimensionally printed thermoplast to a gelatin hydrogel for mechanically enhanced cartilage constructs. Acta Biomater..

[84] Chi J., Wang M., Chen J., Hu L., Chen Z., Backman L.J., Zhang W. (2022). Topographic Orientation of Scaffolds for Tissue Regeneration: Recent Advances in Biomaterial Design and Applications. Biomimetics.

[85] Jeong H.J., Nam H., Jang J., Lee S.J. (2020). 3D Bioprinting Strategies for the Regeneration of Functional Tubular Tissues and Organs. Biomimetics.

[86] Albanna M., Binder K.W., Murphy S.V., Kim J., Qasem S.A., Zhao W., Tan J., El-Amin I.B., Dice D.D., Marco J. (2019). In Situ Bioprinting of Autologous Skin Cells Accelerates Wound Healing of Extensive Excisional Full-Thickness Wounds. Sci. Rep..

[87] Gudapati H., Dey M., Ozbolat I. (2016). A comprehensive review on droplet-based bioprinting: Past, present and future. Biomaterials.

[88] Park J.A., Lee H.R., Park S.Y., Jung S. (2020). Self-Organization of Fibroblast-Laden 3D Collagen Microstructures from Inkjet-Printed Cell Patterns. Adv. Biosyst..

[89] Peng W., Unutmaz D., Ozbolat I.T. (2016). Bioprinting towards Physiologically Relevant Tissue Models for Pharmaceutics. Trends Biotechnol..

[90] Seol Y.-J., Kang H.-W., Lee S.J., Atala A., Yoo J.J. (2014). Bioprinting technology and its applications. Eur. J. Cardio-Thorac. Surg..

[91] Zimmermann R., Hentschel C., Schron F., Moedder D., Buttner T., Atallah P., Wegener T., Gehring T., Howitz S., Freudenberg U. (2019). High resolution bioprinting of multi-component hydrogels. Biofabrication.

[92] Onses M.S., Sutanto E., Ferreira P.M., Alleyne A.G., Rogers J.A. (2015). Mechanisms, Capabilities, and Applications of High-Resolution Electrohydrodynamic Jet Printing. Small.

[93] Liu T., Huang R., Zhong J., Yang Y., Tan Z., Tan W. (2017). Control of cell proliferation in E-jet 3D-printed scaffolds for tissue engineering applications: The influence of the cell alignment angle. J. Mater. Chem. B.

[94] Derby B. (2008). Bioprinting: Inkjet printing proteins and hybrid cell-containing materials and structures. J. Mater. Chem..

[95] Nowicki M., Castro N.J., Rao R., Plesniak M., Zhang L.G. (2017). Integrating three-dimensional printing and nanotechnology for musculoskeletal regeneration. Nanotechnology.

[96] Hrynevich A., Elci B.S., Haigh J.N., McMaster R., Youssef A., Blum C., Blunk T., Hochleitner G., Groll J., Dalton P.D. (2018). Dimension-Based Design of Melt Electrowritten Scaffolds. Small.

[97] Bellehumeur C., Li L., Sun Q., Gu P. (2004). Modeling of Bond Formation Between Polymer Filaments in the Fused Deposition Modeling Process. J. Manuf. Process..

[98] Zielinski P.S., Gudeti P.K.R., Rikmanspoel T., Wlodarczyk-Biegun M.K. (2023). 3D printing of bio-instructive materials: Toward directing the cell. Bioact. Mater..

[99] Liu K., Li W., Chen S., Wen W., Lu L., Liu M., Zhou C., Luo B. (2020). The design, fabrication and evaluation of 3D printed gHNTs/gMgO whiskers/PLLA composite scaffold with honeycomb microstructure for bone tissue engineering. Compos. Part B Eng..

[100] Kade J.C., Dalton P.D. (2021). Polymers for Melt Electrowriting. Adv. Healthc. Mater..

[101] Castilho M., van Mil A., Maher M., Metz C.H.G., Hochleitner G., Groll J., Doevendans P.A., Ito K., Sluijter J.P.G., Malda J. (2018). Melt Electrowriting Allows Tailored Microstructural and Mechanical Design of Scaffolds to Advance Functional Human Myocardial Tissue Formation. Adv. Funct. Mater..

[102] Reneker D.H., Chun I. (1996). Nanometre diameter fibres of polymer, produced by electrospinning. Nanotechnology.

[103] Sun D., Chang C., Li S. (2006). Near-Field Electrospinning. Nano Lett..

[104] Fattahi P., Dover J.T., Brown J.L. (2017). 3D Near-Field Electrospinning of Biomaterial Microfibers with Potential for Blended Microfiber-Cell-Loaded Gel Composite Structures. Adv. Healthc. Mater..

[105] Lin Y., Huang Y., Chrisey D.B. (2011). Metallic foil-assisted laser cell printing. J. Biomech. Eng..

[106] Zhang P., Wang H., Wang P., Zheng Y., Liu L., Hu J., Liu Y., Gao Q., He Y. (2021). Lightweight 3D bioprinting with point by point photocuring. Bioact. Mater..

[107] Antoshin A.A., Churbanov S.N., Minaev N.V., Zhang D., Zhang Y., Shpichka A.I., Timashev P.S. (2019). LIFT-bioprinting, is it worth it?. Bioprinting.

[108] Lewis J.A., Gratson G.M. (2004). Direct writing in three dimensions. Mater. Today.

[109] Yang H., Yang K.H., Narayan R.J., Ma S. (2021). Laser-based bioprinting for multilayer cell patterning in tissue engineering and cancer research. Essays Biochem..

[110] Koch L., Deiwick A., Franke A., Schwanke K., Haverich A., Zweigerdt R., Chichkov B. (2018). Laser bioprinting of human induced pluripotent stem cells-the effect of printing and biomaterials on cell survival, pluripotency, and differentiation. Biofabrication.

[111] Zhang Z., Chai W., Xiong R., Zhou L., Huang Y. (2017). Printing-induced cell injury evaluation during laser printing of 3T3 mouse fibroblasts. Biofabrication.

[112] Sorkio A., Koch L., Koivusalo L., Deiwick A., Miettinen S., Chichkov B., Skottman H. (2018). Human stem cell based corneal tissue mimicking structures using laser-assisted 3D bioprinting and functional bioinks. Biomaterials.

[113] O’Connell C.D., Zhang B., Onofrillo C., Duchi S., Blanchard R., Quigley A., Bourke J., Gambhir S., Kapsa R., Di Bella C. (2018). Tailoring the mechanical properties of gelatin methacryloyl hydrogels through manipulation of the photocrosslinking conditions. Soft Matter.

[114] Kumar H., Kim K. (2020). Stereolithography 3D Bioprinting. Methods Mol. Biol..

[115] Huang J., Qin Q., Wang J. (2020). A Review of Stereolithography: Processes and Systems. Processes.

[116] Lee S.J., Nowicki M., Harris B., Zhang L.G. (2017). Fabrication of a Highly Aligned Neural Scaffold via a Table Top Stereolithography 3D Printing and Electrospinning. Tissue Eng. Part A.

[117] Koons G.L., Diba M., Mikos A.G. (2020). Materials design for bone-tissue engineering. Nat. Rev. Mater..

[118] Oryan A., Alidadi S., Moshiri A., Maffulli N. (2014). Bone regenerative medicine: Classic options, novel strategies, and future directions. J. Orthop. Surg. Res..

[119] Yang Y., Wang G., Liang H., Gao C., Peng S., Shen L., Shuai C. (2019). Additive manufacturing of bone scaffolds. Int. J. Bioprint..

[120] Yu J., Xu Y., Li S., Seifert G.V., Becker M.L. (2017). Three-Dimensional Printing of Nano Hydroxyapatite/Poly(ester urea) Composite Scaffolds with Enhanced Bioactivity. Biomacromolecules.

[121] Nadi A., Khodaei M., Javdani M., Mirzaei S.A., Soleimannejad M., Tayebi L., Asadpour S. (2022). Fabrication of functional and nano-biocomposite scaffolds using strontium-doped bredigite nanoparticles/polycaprolactone/poly lactic acid via 3D printing for bone regeneration. Int. J. Biol. Macromol..

[122] Zhang L., Webster T.J. (2009). Nanotechnology and nanomaterials: Promises for improved tissue regeneration. Nano Today.

[123] Lee S.J., Zhu W., Nowicki M., Lee G., Heo D.N., Kim J., Zuo Y.Y., Zhang L.G. (2018). 3D printing nano conductive multi-walled carbon nanotube scaffolds for nerve regeneration. J. Neural Eng..

[124] Lee S.J., Zhu W., Heyburn L., Nowicki M., Harris B., Zhang L.G. (2017). Development of Novel 3-D Printed Scaffolds With Core-Shell Nanoparticles for Nerve Regeneration. IEEE Trans. Biomed. Eng..

[125] Pi Q., Maharjan S., Yan X., Liu X., Singh B., van Genderen A.M., Robledo-Padilla F., Parra-Saldivar R., Hu N., Jia W. (2018). Digitally Tunable Microfluidic Bioprinting of Multilayered Cannular Tissues. Adv. Mater..

[126] Miao Y., Chen Y., Luo J., Liu X., Yang Q., Shi X., Wang Y. (2023). Black phosphorus nanosheets-enabled DNA hydrogel integrating 3D-printed scaffold for promoting vascularized bone regeneration. Bioact. Mater..

[127] Hann S.Y., Cui H., Esworthy T., Zhou X., Lee S.-J., Plesniak M.W., Zhang L.G. (2021). Dual 3D printing for vascularized bone tissue regeneration. Acta Biomater..

[128] Yang Q., Li J., Su W., Yu L., Li T., Wang Y., Zhang K., Wu Y., Wang L. (2022). Electrospun aligned poly(ε-caprolactone) nanofiber yarns guiding 3D organization of tendon stem/progenitor cells in tenogenic differentiation and tendon repair. Front. Bioeng. Biotechnol..

[129] Wu Y., Wang Z., Fuh J.Y.H., Wong Y.S., Wang W., Thian E.S. (2016). Mechanically-enhanced three-dimensional scaffold with anisotropic morphology for tendon regeneration. J. Mater. Sci. Mater. Med..

[130] Ren Y., Yang X., Ma Z., Sun X., Zhang Y., Li W., Yang H., Qiang L., Yang Z., Liu Y. (2021). Developments and Opportunities for 3D Bioprinted Organoids. Int. J. Bioprinting.

[131] Ding X., Li G., Zhang P., Jin E., Xiao C., Chen X. (2021). Injectable Self-Healing Hydrogel Wound Dressing with Cysteine-Specific On-Demand Dissolution Property Based on Tandem Dynamic Covalent Bonds. Adv. Funct. Mater..

[132] Tasnim N., De la Vega L., Kumar S.A., Abelseth L., Alonzo M., Amereh M., Joddar B., Willerth S.M. (2018). 3D Bioprinting Stem Cell Derived Tissues. Cell. Mol. Bioeng..

[133] Lei Q., He J., Li D. (2019). Electrohydrodynamic 3D printing of layer-specifically oriented, multiscale conductive scaffolds for cardiac tissue engineering. Nanoscale.

[134] Rajendran D., Hussain A., Yip D., Parekh A., Shrirao A., Cho C.H. (2017). Long-term liver-specific functions of hepatocytes in electrospun chitosan nanofiber scaffolds coated with fibronectin. J. Biomed. Mater. Res. Part A.

